# Camouflaging and autism: Conceptualisation and methodological issues

**DOI:** 10.1177/13623613261420085

**Published:** 2026-02-21

**Authors:** Wayne M Arnold, Vicki Bitsika, Christopher F Sharpley

**Affiliations:** 1 Brain-Behaviour Research Group, University of New England, Australia

**Keywords:** autism, camouflaging, conceptualisation, masking, measurement, validity

## Abstract

It has been suggested that there is poor clarity of the ‘camouflaging’ concept in autism research, and potential confounding of its measurement tools, such as the Camouflaging Autistic Traits Questionnaire (CAT-Q). A critical review of 389 studies was conducted to investigate these potential conceptual and methodological issues. The findings question whether the same construct has been investigated across studies, as there is inconsistency in: (a) which terms are used; (b) reference made to established conceptual literature; (c) how terms are used (e.g. interchangeably or distinctly); and (d) how terms are defined. Although the CAT-Q has excellent reliability, there is mixed support for its validity, which is evidenced by its confounding by other constructs (e.g. social anxiety) and its limited autism-specificity. The validity of informant discrepancy measures of camouflaging is also questioned due to insufficient reference to established methodology. Finally, the generalisability of camouflaging to the overall autistic population is unclear due to: (a) overrepresentation of autistic females diagnosed during adulthood; and (b) underrepresentation of autistic people with co-occurring intellectual or language difficulties, and those with greater support needs. These issues are considered both in terms of their clinical relevance and how future research might resolve them.

**Lay Abstract:**

Many autistic people have reported using ‘camouflaging’ strategies to adapt or cope within the non-autistic social world and avoid being negatively judged by other people. However, many terms have been used synonymously with camouflaging, such as masking, compensation and impression management. Due to this confusion about which terms to use, there is some suggestion that there is poor clarity and understanding of the camouflaging concept, and that this may contribute to inaccuracy of the tools used to measure this behaviour. We review 389 previous studies to examine these concerns. Our findings confirm this lack of clarity by showing that studies are inconsistent in: (a) which terms they used to refer to behavioural strategies that resemble camouflaging; (b) whether they referred to existing literature; (c) whether they used different terms to refer to the same concept or to separate types of behaviour; and (d) how they defined the terms that they used. Our findings also question the accuracy of camouflaging measurement tools, as these tools may also be measuring other behaviours (e.g. social anxiety) that are not only experienced by autistic people. We also find that camouflaging studies have mostly focused on autistic females with no accompanying cognitive or language difficulties, and who have received their diagnosis in adulthood. Although camouflaging may contribute to the underdiagnosis of some autistic females, most autistic people are male and are diagnosed during early childhood, and a large number of autistic people do experience those other difficulties. Because of this, it is not clear whether the findings of previous camouflaging studies can apply to all autistic people. We provide some suggestions for how researchers might resolve some of these issues in their future studies, and we consider what the findings mean for clinicians who work with autistic people.

Current diagnostic processes for autism involve clinician observations and caregiver-derived information on course of development (American Psychiatric Association [APA], 2022). If behaviour consistent with an autism diagnosis is not evident, this is likely to impact diagnostic identification. For instance, if an autistic individual ‘camouflages’^1^ their autistic traits, then they may be undetected or misdiagnosed during this assessment process, or not referred for assessment in the first instance; thus, camouflaging is a barrier to timely and accurate diagnosis of autism (Lai & Baron-Cohen, 2015; Lai et al., 2022).

One prominent definition suggests camouflaging broadly involves “the employment of specific behavioural and cognitive strategies by autistic people to adapt to or cope within the predominantly non-autistic social world” (Cook, Hull, et al., 2021, p. 1). Specific categories of camouflaging are suggested to include: (a) ‘compensation’ for autism-related social differences by employing structured techniques to navigate social interactions; (b) ‘masking’ by hiding one’s autistic traits and/or presenting a non-autistic persona; and (c) ‘assimilation’ strategies that facilitate blending in with others in social interactions (Hull et al., 2017, 2019). Autistic people may camouflage to ‘fit in’ with other people in social settings, or to avoid social rejection that may occur due to stigma (Ai et al., 2022; W. B. Lawson, 2020; Pearson & Rose, 2021; Radulski, 2022), which refers to negative stereotypes, attitudes and beliefs held about autism and its behavioural traits that may lead to experiences of discrimination and prejudice (Link & Phelan, 2001). Because these social integration and stigma avoidance motives are pervasive across social settings, camouflaging may become habitual and employed automatically without conscious awareness (Field et al., 2024; W. B. Lawson, 2020).

Camouflaging has predominantly been measured quantitatively via two methods. First, the informant discrepancy method, which operationalises camouflaging as the statistical discrepancy between two informant measures of autistic behaviour: (a) observer-report (e.g. the Autism Diagnostic Observation Schedule; Lord et al., 2000); and (b) self-report (e.g. the Autism Spectrum Quotient; Baron-Cohen et al., 2001). This informant discrepancy is thought to represent a mismatch between the ‘external’ observed, and ‘internal’ self-reported autistic presentation (Lai et al., 2017). The second method is the Camouflaging Autistic Traits Questionnaire (CAT-Q; Hull et al., 2019), a psychometric scale that operationalises camouflaging as consisting of three subcomponents: ‘compensation’, ‘masking’ and ‘assimilation’ (defined above). Some other camouflaging measures have also been developed, but these are not widely used, and research into their validity is ongoing. These include the ‘Masking’ subscale of the Comprehensive Autistic Trait Inventory (CATI; English et al., 2021; Hechler et al., 2025), the Compensation Checklist (Livingston et al., 2020), and experimental methods wherein camouflaging is inferred following observation of autistic people’s social interaction behaviours (e.g. Dean et al., 2017).

Despite these developments, camouflaging remains poorly understood, often due to inconsistencies in how it is defined. For example, there is limited consensus about which terms should be used when describing camouflaging behaviour (Fombonne, 2020; Lai et al., 2020; Libsack et al., 2021), and this has supported a need for greater conceptual clarity (Cremone et al., 2023; Fombonne, 2020; Lai et al., 2020; Schuck et al., 2019). Furthermore, because psychometric tools rely on clarity of the underlying constructs being measured (Bringmann et al., 2022; MacKenzie, 2003), disagreement about the nature of camouflaging has potentially contributed to confounding of these tools, as the CAT-Q appears to measure constructs other than camouflaging (e.g. social anxiety; Fombonne, 2020; Lai et al., 2020).

Increasing conceptual clarity is suggested to first involve reviewing: (a) ambiguities in how camouflaging has been termed and defined, and (b) methodological issues that may have resulted from these ambiguities or otherwise impact the validity of camouflaging research (Bringmann et al., 2022; Racine, 2015). Following this, conceptual analysis may be used to resolve these ambiguities, and study designs and measurement tools can then be adapted to align with the clarified concepts and address the methodological limitations (Jaakkola, 2020; Podsakoff et al., 2016). In accordance with that first stage of increasing clarity, the present review aims to critically appraise the camouflaging literature and identify conceptual and methodological issues that are pervasive across studies.

## Method

Due to inconsistent terminology used in camouflaging research (Fombonne, 2020; Lai et al., 2020; Libsack et al., 2021), a standard literature search process would not identify all relevant studies without *a priori* knowledge of all the terms used synonymously with ‘camouflaging’. An alternative approach, backwards citation searching, performs similarly to standard systematic literature searching in identifying relevant articles, but also facilitates the identification of studies using terms that are not captured by standard search methods (Haddaway et al., 2022; Jalali & Wohlin, 2012). Therefore, following an initial literature search of Google Scholar using the terms ‘autism’ AND ‘camouflaging’ OR ‘masking’ OR ‘compensation’, the search strategy relied primarily on backwards citation searching to identify novel terms that had been used in reference to camouflaging behaviours. Studies were included if they referred to behaviours consistent with the conceptual definition and categories of camouflaging described above. Due to a broad focus of this review on how these behaviours were conceptualised, studies were included even if they only referred to them in passing, irrespective of their study design. Studies were excluded if they referred to non-camouflaging concepts with similar terminology (e.g. monetary ‘compensation’ of study participants), were not published in peer-reviewed journals, and/or were not published in English. This process, completed on 17 March 2025, led to the inclusion of 389 studies, published between 1 January 2000 and 13 March 2025 (see Supplemental Figure S1 for a PRISMA flow diagram). All included studies were analysed according to how they conceptualised camouflaging, with a focus on analysing inconsistencies (e.g. in terminology and definitions) across studies. Methodological limitations (e.g. in study design, sample representativeness, and operationalisation/measurement of camouflaging) that were pervasive across qualitative and/or quantitative studies of camouflaging were also analysed. These conceptual and methodological issues were summarised using narrative synthesis. Data supporting this review are available within the article and supplementary materials.

### Positionality statement

The first author is autistic and was the main contributor to all aspects of the study (conceptualising the review, performing the literature search and formal analysis, interpreting the findings and formulating the conclusions of the review, and co-authoring the article). Because of this, the first author’s lived experiences, as an autistic person who often camouflages, influenced his interpretation of some of the identified conceptual and methodological issues.

## Results

### Conceptual issues

The analysis revealed four forms of inconsistency in how camouflaging was conceptualised across studies. These included inconsistencies in: (a) which terms were used, (b) whether terms were informed by established conceptual literature, (c) how terms were applied (e.g. whether they were interchangeable or distinct concepts), and (d) how terms were defined.

First, as mentioned elsewhere (Fombonne, 2020; Lai et al., 2020; Libsack et al., 2021), considerable heterogeneity was detected in the terminology used, as shown by the list of terms presented in Table 1. Second, as seen in Table 1, the terms are categorised based on whether they are: (a) an established term from existing conceptual literature, or (b) novel terms that have primarily emerged from recent camouflaging research. This demonstrates that camouflaging studies used a mixture of established and novel terms, suggesting inconsistency in referring to established literature.

**Table 1.** List of terms used in autism camouflaging literature.

Established terms	Novel terms
Adaptation, Assimilation, Blending in, Codeswitching, Compensation (strategies), Compensatory learning, (Self-) Concealment, Coping (strategies), Covering (up), (Qualified) Deception, Delayed maturation, Disability management, False self, Fitting in, Identity management, Imitation, Impression management, Invisibility, Mimicry, Modelling, Negotiating difference, Non-disclosure, Observational learning, Over-adaptation, Passing (as non-autistic/neurotypical), Phantom normalcy, (Genuine) Remediation, Reputation management, Self-presentation, Social strategies, Substitution, Suppression	Adaptive morphing, Blended phenotype, Camouflaging (efficacy/intent), Faking neurotypical, Hiding autism, Innocuous engagement, Masking, Masquerading, Neurotypical communication modelling, Normal façade, Pretending to be normal, Social morphing, Transmuting

Variations on terms are presented in parentheses. Established terms are those commonly used in existing domains of psychological and/or neuroscientific literature to describe behavioural change in social settings or across developmental periods, which have been applied to explain findings in autism camouflaging research. This was determined by: (a) examining references made to established conceptual literature in the reviewed studies; plus (b) searching Google Scholar for each term alongside the ‘-autism’ keyword and analysing the search results with respect to whether terms are applied to the study of camouflaging-like behaviour in other populations. Novel terms are those that have emerged primarily from autism camouflaging studies.

Third, in the 389 studies analysed, there was variability in whether: (a) terms were used interchangeably to represent a single underlying behavioural phenomenon (i.e., not as distinct categories of camouflaging; 59.1% of studies); (b) separate terms were used to represent distinct categories of behaviour (19.0%); (c) one term was used to represent a single behavioural phenomenon (19.0%); or (d) in some cases where conceptual confusion was clearly evident, the same term was used both interchangeably and distinctly (2.8%). Supplemental Table S1 lists the studies that fell into each of these four categories, and it is apparent from this analysis that the aforementioned heterogeneity in terms, plus inconsistent references made to established conceptual literature, has led to inconsistency in the applications of those terms (i.e. the conceptualisation, operationalisation and empirical investigation of camouflaging). Despite the CAT-Q being the dominant measurement tool, only 15.9% of studies used a similar conceptualisation (i.e. distinct subcomponents of ‘camouflaging’). Instead, most studies (59.1%) used several terms interchangeably.

Fourth, studies were inconsistent in how they defined camouflaging. For example, some studies did not explicitly define camouflaging when referencing the construct (Allely, 2019; Backer van Ommeren et al., 2017; Ferri et al., 2018; L. P. Lawson et al., 2018; Mademtzi et al., 2018; Moseley et al., 2018). Others provided vague definitions that lacked precision, such as defining camouflaging as (a) a set of general coping strategies (Murphy et al., 2022); (b) the ways in which everyone tries to disguise undesirable aspects of their personality (Dell’Osso et al., 2021); or (c) how autistic females are able “to adapt better socio-communicatively” (Lehnhardt et al., 2016, p. 146).

One explanation for these four conceptual inconsistencies could be that researchers have neglected to distinguish between autism-specific and autism-nonspecific behaviours (Fombonne, 2020; Lai et al., 2020). For instance, one construct that has regularly been considered as conceptually equivalent to camouflaging is impression management/self-presentation. Schlenker (2012) defined ‘impression management’ as “the goal-directed activity of controlling information in order to influence the impressions formed by an audience” (p. 542), while ‘self-presentation’ is the self-directed form of impression management by which “people try to control impressions of themselves, as opposed to other people or entities” (p. 542). In qualitative studies, behaviours described as camouflaging/masking included: imitation, acting and smiling more (Tierney et al., 2016); smiling or making jokes (Cook, Crane, Bourne, et al., 2021; Cook, Crane, Hull et al., 2021); displays of affection (Okamoto et al., 2024); and the concealment of anxious (e.g. Anderson et al., 2020) or depressive (Rhodes et al., 2023) behaviour. These behaviours, rather than indicating reductions in the appearance of autistic traits or autistic identity *per se*, instead show that autistic people reportedly engage in self-presentation (i.e. enacting a false or misleading persona).

Ai et al. (2022) distinguished between two forms of self-presentation observed in autistic people: socially motivated ‘voluntary self-enhancement’, and ‘compelled social modification’, such that the latter was equivalent to a construct known in the wider literature as concealment, a behavioural outcome of stigma (e.g. Pachankis, 2007). Many researchers have similarly equated camouflaging with concealment (Botha & Gillespie-Lynch, 2022; Cage et al., 2022; Cleary et al., 2023; Cook et al., 2023; Durben, 2024; Han et al., 2022; Khudiakova, Alexandrovsky, et al., 2024; Libsack et al., 2021; Loo et al., 2023; McQuaid et al., 2023; Pearson & Rose, 2021; Radulski, 2022; Rivera & Bennetto, 2023; Schneid & Raz, 2020; Shalit et al., 2024; Somerville et al., 2023; Turnock et al., 2022; Zhuang et al., 2023). However, little is known about the overlap between camouflaging findings and existing stigma theory, and research into the distinctions between camouflaging and self-presentation is ongoing (Khudiakova, Alexandrovsky, et al., 2024).

Another autism-nonspecific construct that may also have conceptual similarities to some camouflaging behaviours is social learning. The CAT-Q comprises a ‘compensation’ subscale, tapping behaviours resembling rehearsal, imitation and modelling strategies that are equivalent to social skills acquisition strategies that: (a) are employed during neurotypical child and adolescent development (Frith & Frith, 2012; Ladd, 2005); and (b) occur following observation of others’ social behaviour and its consequences, according to classical social learning and social cognitive theories (Bandura, 1977, 1986). Such behaviours are also routinely taught in structured clinical interventions such as social skills training (Ladd & Mize, 1983), which has efficacy in improving the social competency of many young autistic people (Erhard et al., 2024; Gunning et al., 2019).^2^

Therefore, many so-called ‘compensation’ strategies may instead reflect methods by which autistic people are genuinely learning and applying social skills, rather than ‘compensating’ for their autism-related social differences *per se*. One issue with the term ‘compensation’ is that it may endorse a deficit model of autism by implying that there is a baseline of dysfunction that must be circumvented, as opposed to considering autistic traits as alternative means of perceiving and interacting with the world that may be associated not only with specific needs but also strengths (Bottema-Beutel et al., 2020; Petrolini et al., 2023). Along similar lines, Marocchini (2023) suggested that the use of the term ‘compensation’ amounts to ‘ableism’ (devaluation and discrimination of autistic individuals relative to neurotypical individuals; Smith, 2016) because behavioural indicators of social or cognitive competence (i.e. ‘typical’ behaviour, such as smiling or making jokes, as described above) that autistic people display are categorised as an alternative category of ‘atypical’ autistic behaviour, and that these are distinct from normative social behaviours or skills acquisition processes. Alternatively, the intentional and persistent use of these strategies by autistic people well into adulthood may stem from autism-related neurodevelopmental delays in their social skills acquisition. Therefore, the autism-specificity of these strategies might instead be determined by their extended duration of use across developmental periods, rather than by their behavioural indicators *per se*. This possibility of ‘delayed maturation’ was similarly proposed by Livingston and Happé (2017), but more research is needed on this concept.

### Methodological issues

Methodological issues in camouflaging research were also identified. These included limitations in the: (a) construct validity and (b) autism-specificity of the CAT-Q; (c) validity of informant discrepancy methods due to insufficient reference to established methodological literature; and (d) generalisability of samples employed in empirical studies.

#### Construct validity of the CAT-Q

Across studies that reported them, reliability metrics (internal consistency and test–retest reliability; that is, stability of scores across individuals and time) of the CAT-Q were consistently good to excellent (e.g. Hull et al., 2019). However, reliability does not guarantee that a scale is validly measuring its intended construct (i.e. ‘camouflaging’; DeVellis & Thorpe, 2022; Nunnally & Bernstein, 1994). This section examines whether this is the case by reviewing evidence for the construct validity of the CAT-Q.

In the validation study of the CAT-Q, measures of autistic traits, wellbeing, social and generalised anxiety, and depression were utilised as indicators of convergent validity (Hull et al., 2019). However, correlations with external variables such as these are not indicative of convergent validity, but of criterion validity; a more appropriate measure of convergent validity would be another measure of camouflaging (Nunnally & Bernstein, 1994). This is therefore determined by correspondence between the CAT-Q and informant discrepancy methods, but these measures are weakly correlated (Hannon et al., 2022; van der Putten et al., 2023), and they disagree in supporting sex/gender differences in camouflaging (Cook, Hull, et al., 2021). Similarly, a parent-report version of the CAT-Q was found not to correlate strongly with the self-report version, and so these were not “considered [as] fully interchangeable” (Hannon et al., 2022, p. 13). Because of this limited convergent validity, camouflaging measures may reflect “different facets or features of camouflaging” (Hannon et al., 2022, p. 13). For example, self-report measures may reflect camouflaging ‘intent’ (i.e. the self-reported intention to engage in camouflaging), while discrepancy variables may represent camouflaging ‘efficacy’ (i.e. the efficacy of camouflaging in real-world social interactions) (Cook, Hull, et al., 2021; Williams, 2022). More research is needed to support these nuanced distinctions.

Evidence for the criterion validity of the CAT-Q is also mixed. An association between the CAT-Q and poorer mental health is generally supported (as reviewed by Khudiakova, Russell, et al., 2024). However, the scale weakly correlates with autistic burnout (Arnold et al., 2023b; Mantzalas et al., 2024), and there is mixed evidence for associations with diagnostic delay (Belcher et al., 2021; Gonçalves Garcia et al., 2025; Milner et al., 2023) and stigma (Keating et al., 2024; Perry et al., 2021; Zhuang et al., 2024). These findings contradict reviews and theory on the correlates of camouflaging that are informed by qualitative research (Arnold et al., 2023a; Cook et al., 2024; Higgins et al., 2021; Huang et al., 2020; Lai & Baron-Cohen, 2015; Lai et al., 2022; Lockwood Estrin et al., 2021; Mantzalas, Richdale and Dissanayake, 2022; Pearson & Rose, 2021; Zhuang et al., 2023), suggesting that autistic people’s qualitative experiences of camouflaging may not directly correspond with the quantitative measurement of camouflaging using the CAT-Q.

In addition, the CAT-Q’s masking subscale has limited content validity, as not all aspects of ‘masking’ are measured (Clark & Watson, 2019; Nunnally & Bernstein, 1994). A review by Collis et al. (2024) found that restricted and repetitive behavioural autistic traits (RRBs) are masked by autistic people, including behavioural indicators of sensory overload (overstimulation resulting from sensory hypersensitivity) and stimming (self-stimulatory behaviours which can regulate stress levels, among other functions; Charlton et al., 2021; Kapp et al., 2019). Masking of these RRBs is widely reported in qualitative research (Chapman et al., 2022; Cook, Crane, Hull et al., 2021; Finn et al., 2023; Hull et al., 2017; Livingston, Shah, & Happé, 2019; Milner et al., 2019; Tint & Weiss, 2018), but the CAT-Q’s masking subscale does not capture this, which may be because the CAT-Q focuses exclusively on the camouflaging of social autistic traits and not also on RRBs.

A final consideration relating to the construct validity of the CAT-Q is the replicability of its three-factor structure (DeVellis & Thorpe, 2022; Nunnally & Bernstein, 1994; Osborne & Fitzpatrick, 2012). This factor structure was replicated in a large general population sample of the United States (Ai et al., 2023), and also with a short-form version, which correlated strongly with the original full-length scale (Hull et al., 2024). However, Dutch, Japanese, Swedish, and traditional Chinese translations of the CAT-Q could not replicate its three-factor structure (Hongo et al., 2024; Liu et al., 2023; Remnélius & Bölte, 2023; van der Putten et al., 2023). Furthermore, the Dutch and French versions failed to demonstrate measurement invariance, meaning that autistic and non-autistic groups were not comparable in their scale scores (Bureau et al., 2023; van der Putten et al., 2023). Failure to replicate the CAT-Q’s factor structure could be due to variability in the nature of camouflaging across cultures (Keating et al., 2024; Khudiakova, Alexandrovsky, et al., 2024; Loo et al., 2023; Oshima et al., 2024; Remnélius & Bölte, 2023), but more research is needed to determine this.

#### Autism-specificity of the CAT-Q and its subscales

Several findings also question whether the behaviours tapped by the CAT-Q are specific to the experiences of autistic people. These include: (a) correlations between the CAT-Q and autistic traits are often weaker than with traits indicative of other conditions; (b) the masking subscale is unrelated to autistic traits, and poorly differentiates between autistic and non-autistic groups; and (c) potential confounding of the CAT-Q by constructs that are not autism-specific.

First, the CAT-Q was more strongly related to attention-deficit/hyperactivity disorder (ADHD) traits than autistic traits in one study (Ai et al., 2023), while adults with ADHD engaged in significantly greater camouflaging than a neurotypical comparison group in another study (van der Putten et al., 2024). Furthermore, total CAT-Q scores correlated more strongly with social anxiety than with autistic traits in the scale’s validation study (Hull et al., 2019). This finding has since been replicated in seven other studies (Hull et al., 2021; Lei et al., 2024; Lorenz & Hull, 2024; McKinnon et al., 2024; Porricelli et al., 2024; Pyszkowska, 2024; Tamura et al., 2024), while Galvin et al. (2024) found that these two constructs correlated with the CAT-Q to a similar degree. Only one study observed a stronger correlation between the CAT-Q and autistic traits than with social anxiety (Oshima et al., 2024). Social anxiety is elevated in autistic people relative to neurotypical people (Spain et al., 2018), and this might explain why the former often score higher on the CAT-Q than the latter (Cook, Hull, et al., 2021).

A second category of findings that question the autism-specificity of the CAT-Q relates to its masking subscale. Contradicting its operational definition (“hiding aspects of one’s autistic presentation, or presenting a non-autistic persona to others”, Hull et al., 2019, p. 825), this subscale has been widely found to be unrelated to autistic traits (Han et al., 2022; Hongo et al., 2024; Hull et al., 2019, 2024; Kong et al., 2024; Lei et al., 2024; McKinnon et al., 2024; Nieradka & Kossewska, 2023; Remnélius & Bölte, 2023, in the adolescent subsample; van der Putten et al., 2024). Similarly, with respect to effect sizes, the subscale also weakly differentiated between: (a) autistic and non-autistic adults in four studies (Hongo et al., 2024; Hull et al., 2019, 2024; Hull, Lai, et al., 2020), and (b) neurodivergent and neurotypical adolescent girls (McKinney et al., 2024). In contrast, non-autistic adolescents scored significantly higher in masking than autistic adolescents in two studies (Jorgenson et al., 2020; Lei et al., 2024).

Third, many of the CAT-Q’s items appear to be confounded by constructs that are not autism-specific. As discussed previously, many ‘compensation’ items resemble social learning strategies employed during typical social development (Bandura, 1977, 1986; Frith & Frith, 2012), suggesting confounding. Other behavioural rehearsal strategies tapped by this subscale, such as the use of ‘social scripts’ (e.g. “I have developed a script to follow in social situations (for example, a list of questions or topics of conversation)”, Hull et al., 2019, p. 827), share conceptual similarities with the ‘backstage’ of self-presentation behaviour, during which enacted personas are privately cultivated and rehearsed (Goffman, 1959). This is supported by a strong positive correlation between the Self-Presentation Tactics scale and the CAT-Q (Ai et al., 2023).

Similarly, most items on the ‘masking’ subscale appear to tap into self-monitoring (McKinnon et al., 2024), which refers to a disposition towards active monitoring of the social environment, such as attending to social cues (e.g. other people’s reactions) that might inform the appropriateness of one’s social behaviour (Snyder, 1974). This confounding is supported by correlations between masking and public self-consciousness and self-presentation (Ai et al., 2023; Lei et al., 2024), which are closely related to self-monitoring (Gangestad & Snyder, 2000). It is suggested that self-monitoring the visibility of one’s autistic traits in social settings is a precursor to ‘masking’ them (Ai et al., 2022; Radulski, 2022), so it is unlikely that self-monitoring is a masking strategy *per se*, but rather a personality dimension/learnt behaviour that facilitates this. Only three of the masking items strictly adhere to its operational definition (provided above). One item pertains to hiding atypical patterns of eye contact (“I don’t feel the need to make eye contact with other people if I don’t want to”, Hull et al., 2019, p. 827; item is reverse-keyed), but this item was dropped from the scale in four studies due to poor factor loading (Ai et al., 2023; Hongo et al., 2024; Liu et al., 2023; van der Putten et al., 2023), suggesting that its content is inconsistent with the other masking items. Two other items measure reductions in tenseness (“I adjust my body language or facial expressions so that I appear relaxed”, Hull et al., 2019, p. 827) and social disinterest (“I adjust my body language or facial expressions so that I appear interested by the person I am interacting with”, Hull et al., 2019, p. 827) in social settings, which may relate to social autistic traits (APA, 2022) but might also be explained by social anxiety (Fombonne, 2020).

Finally, the ‘assimilation’ items of the CAT-Q are confounded by social anxiety, demonstrated by significant construct overlap in two factor analytic studies (Lei et al., 2024; McKinnon et al., 2024). Collectively, these data suggest that many of the behaviours tapped by the CAT-Q are not unique to the experiences of autistic people, contradicting its intention to measure behaviours specifically engaged in by autistic people (Hull et al., 2019). There is currently no consensus on how camouflaging is distinct from social learning, self-presentation, self-monitoring, or social anxiety, and there was no distinction made between these constructs and camouflaging during the development of the CAT-Q, leaving the scale open to confounding (Khudiakova, Alexandrovsky, et al., 2024).

#### Validity of informant discrepancy measurement of camouflaging

Similar to the CAT-Q, Fombonne (2020) suggested that informant discrepancy measures of camouflaging are open to confounding. Specifically, informant discrepancy measures assume that higher scores of self-reported autistic traits are a more valid indicator of overt autistic behaviour than observer-reported autistic traits in the presence of camouflaging. However, this statistical discrepancy may instead reflect measurement error in estimating the same underlying latent construct (i.e. autistic behaviour), and it is also unclear that this approach is uniquely measuring camouflaging *per se*. Adding to this perspective, discrepancy-based camouflaging studies have universally ignored the available methodological literature, which aims to differentiate discrepancy variables from measurement error or from confounding constructs (e.g. De Los Reyes et al., 2013). Rather than camouflaging, a statistical discrepancy between self and observer ratings of autistic behaviour may reflect other means of behavioural modification while being observed that may not be specific to autistic traits (e.g. self-presentation or socially desirable responding; De Los Reyes et al., 2015). This was demonstrated in two studies of females with ADHD, where informant discrepancies were correlated with self-presentation (Johnston et al., 2004; Ohan & Johnston, 2011). Greater reference to established methods is needed to ensure that informant discrepancy measures are validly measuring camouflaging.

In addition, camouflaging studies are inconsistent in their selection of self-report variables used to calculate informant discrepancies. Self-report variables included (a) self-reported (Milner et al., 2022; Schuck et al., 2019) or parent-reported (Ross et al., 2022) autistic traits; (b) theory of mind (Corbett et al., 2020; Livingston, Colvert, et al., 2019; Milner et al., 2022; Wood-Downie et al., 2020); or (c) composite measures of both autistic traits and theory of mind (Lai et al., 2017, 2019; Trakoshis et al., 2020). More consistency is therefore needed across discrepancy studies, such as by selecting discrepancy variables that maximise their convergent validity with self-report measures of camouflaging.

#### Generalisability of camouflaging

A final methodological issue revealed by this review is that the samples employed in camouflaging studies have limited generalisability to the wider autistic population (Petrolini et al., 2023), in which three to four males are diagnosed for every female (Loomes et al., 2017; Zeidan et al., 2022), and the global average age at diagnosis is 60 months (van’t Hof et al., 2020). Instead, most studies have employed samples of predominantly, or exclusively, autistic females diagnosed during adulthood (Baldwin & Costley, 2016; Bargiela et al., 2016; L. Bradley et al., 2021; Brake, 2024; Cage et al., 2018; Cage & Troxell-Whitman, 2019; Cook, Crane, Hull et al., 2021; Cooper, 2024; Craddock, 2024; Hull et al., 2017, 2019; Leedham et al., 2020; Livingston, Shah, & Happé, 2019; Murphy et al., 2022; Rynkiewicz et al., 2016; Seers & Hogg, 2022; Tierney et al., 2016; Tint & Weiss, 2018). If autistic females camouflage more than autistic males, then this may contribute to their relative underdiagnosis, and therefore to inaccurate population estimates of gender ratios in autism diagnosis (Cook et al., 2024; Cruz et al., 2024). However, despite this overreliance on sampling autistic females, there is limited evidence of sex/gender differences in camouflaging (Cook, Hull, et al., 2021). Furthermore, the conceptual distinction between camouflaging and other sex/gender-nonspecific constructs, such as self-presentation and concealment, remains unclear. This suggests that camouflaging may not be applicable only to females, and should not form the basis of a female autism phenotype (Lai et al., 2020, 2022; Pearson & Rose, 2021).

In addition, undiagnosed individuals, who self-identify as autistic or who exceed a clinical threshold of autistic traits, need to be sampled to fully understand the extent of camouflaging and its potential impacts on diagnostic reliability (Hull et al., 2019; Lai & Baron-Cohen, 2015). However, a minority of quantitative (S. Bradley et al., 2024; Cage et al., 2022; Cage & Troxell-Whitman, 2020; Evans et al., 2023; Graham et al., 2023; Moore et al., 2023; Oshima et al., 2024; Robinson et al., 2020), qualitative (Angulo et al., 2019; Charlton et al., 2021; Gemma, 2021; Harmens et al., 2022; Lewis & Stevens, 2023; Livingston, Shah, & Happé, 2019; Mantzalas, Richdale, Adikari et al., 2022; Miller et al., 2021; Milner et al., 2019; Mosquera et al., 2021; Pearson et al., 2022; Pryke-Hobbes et al., 2023; Schneid & Raz, 2020) and mixed methods (L. Bradley et al., 2021; Wiskerke et al., 2018) studies have included such people. Similarly, intellectual disability co-occurs in 33% of the autistic population (Zeidan et al., 2022), yet camouflaging studies have routinely excluded this subpopulation (Petrolini et al., 2023). Also excluded have been those with greater support needs in domains of daily functioning, and those with co-occurring cognitive or language difficulties (Libsack et al., 2021; Petrolini et al., 2023). More research is therefore needed into how camouflaging might be relevant, or how it may differ in its presentation, for these understudied autistic subpopulations.

## Discussion

The findings of this review align with previous concerns about the clarity and validity of researchers’ current conceptualisations of ‘camouflaging’. Conceptual inconsistencies were identified in: (a) the terms used to describe camouflaging, (b) reference to established literature on similar behavioural constructs studied in other populations, (c) how terms were applied (e.g. to their operationalisation and empirical study), and (d) how terms were defined. Four methodological issues were also identified, namely: (a) limited construct validity and (b) autism-specificity of the CAT-Q, (c) limited validity of informant discrepancy measures, and (d) unclear generalisability of camouflaging to the wider autistic population.

### Strengths and limitations

It is important to recognise some strengths and limitations of the present review. Due to inconsistent terminology in camouflaging research, a highly inclusive, albeit unsystematic approach to literature searching and study inclusion was used, primarily via the use of backwards citation searching. Studies were then summarised using narrative synthesis. This approach was limited in that: (a) qualitative/quantitative studies that were analysed for the purpose of identifying methodological issues were not assessed for their quality, which may have influenced the nature of the identified issues; (b) the findings of each study were not described in detail; (c) a single database was searched to identify the initial pool of articles prior to backwards citation searching, which may have limited the comprehensiveness of article inclusion; and (d) there may be some risk of bias in the data synthesis and interpretation procedures due to the non-systematic approach used. However, one strength of this approach is that many terms were identified that were not used in the search strategies of previous camouflaging reviews (Alaghband-Rad et al., 2023; Cook, Hull, et al., 2021; Cruz et al., 2024; Field et al., 2024; Han et al., 2022; Hull, Petrides, et al., 2020; Khudiakova, Russell, et al., 2024; Klein et al., 2024; Libsack et al., 2021; Ridgway et al., 2024; Tubío-Fungueiriño et al., 2020; Zhuang et al., 2023) due to those reviews often restricting searches to the most commonly used terms (i.e. camouflaging, masking, and compensation). This finding of additional terminology might enable future reviews of these behaviours to be more comprehensive in their literature search and study inclusion procedures.

### Future directions

There are several ways that researchers might address the issues identified in this review. First, in addressing the conceptual inconsistencies, researchers are suggested to conduct additional conceptual analyses. For example, some researchers have considered camouflaging through a lens of self-presentation/impression management (Ai et al., 2022; Khudiakova, Alexandrovsky, et al., 2024). However, there is limited synthesis with existing stigma theory (Durben, 2024; Turnock et al., 2022), despite researchers commonly equating aspects of camouflaging with concealment, a behavioural outcome of stigma (Botha & Gillespie-Lynch, 2022; Cage et al., 2022; Cleary et al., 2023; Cook et al., 2023; Durben, 2024; Han et al., 2022; Khudiakova, Alexandrovsky, et al., 2024; Libsack et al., 2021; Loo et al., 2023; McQuaid et al., 2023; Pearson & Rose, 2021; Radulski, 2022; Rivera & Bennetto, 2023; Schneid & Raz, 2020; Shalit et al., 2024; Somerville et al., 2023; Turnock et al., 2022; Zhuang et al., 2023). Camouflaging has similarities with several other behavioural concepts (e.g. self-presentation, concealment, social anxiety, self-monitoring, and social learning), and so more research is needed to inform the distinctions between these.

Furthermore, these distinctions were not made during the development of the CAT-Q, leaving the scale open to confounding (Khudiakova, Alexandrovsky, et al., 2024). The findings of the present review suggest that this confounding could explain mixed psychometric support for the CAT-Q, as well as inconsistent sex/gender differences in camouflaging. Greater refinement of camouflaging measurement is therefore needed, particularly tools that are informed by a more robust conceptual foundation (Khudiakova, Alexandrovsky, et al., 2024; Khudiakova, Russell, et al., 2024; Lai et al., 2020). For instance, scales that separately measure self-presentation, concealment, or social learning processes in autistic people may be necessary. Scales also should more comprehensively tap into the camouflaging of non-social autistic traits (e.g. suppressing stimming), as the CAT-Q is limited in this aspect of content validity. More research is also needed to establish whether camouflaging measures are able to tap into unconscious/automatic camouflaging, which results from its habitual engagement across social settings (Field et al., 2024; W. B. Lawson, 2020). Finally, there is a need to further validate other camouflaging measures that are not yet widely used, such as the CATI (English et al., 2021; Hechler et al., 2025), the Compensation Checklist (Livingston et al., 2020) and experimental methods (e.g. Dean et al., 2017).

In addition, established methods should be adhered to when using informant discrepancies to measure camouflaging, such as those described in publications by Andres De Los Reyes and colleagues (De Los Reyes, 2011, 2013; De Los Reyes et al., 2013, 2015; Laird & De Los Reyes, 2013). For example, the Operations Triad Model (De Los Reyes et al., 2013) offers a systematic approach to differentiate valid discrepancy variables (i.e. ‘Diverging Operations’) from those reflective of measurement error or other sources of confounding (i.e. ‘Compensating Operations’). Specifically, some statistical methods (e.g. structural equation modelling, polynomial regression) may be used to partition out shared versus unique variance between informant ratings to differentiate these discrepancies from measurement error (De Los Reyes et al., 2013; Laird & De Los Reyes, 2013). Furthermore, associations between informant discrepancies and potential confounding constructs (e.g. cognitive ability of raters, social desirability, and/or self-presentation) must be ruled out (De Los Reyes et al., 2015). Finally, more research is needed to investigate the validity of the differing variables used to calculate informant discrepancies, as studies were inconsistent in their selection of self-report variables (e.g. autistic traits, theory of mind). Following these methodological improvements, the convergent validity of informant discrepancy and self-report measures of camouflaging should be investigated to ensure that these methods are equivalent in measuring camouflaging. If they are not equivalent, then it may be that these methods measure differing aspects of camouflaging (e.g. intent vs. efficacy; Cook, Hull, et al., 2021; Williams, 2022).

A final consideration for researchers is that the generalisability of camouflaging concepts to the wider autistic population has not been established, because samples have: (a) seldom corresponded with population estimates of sex/gender ratios and age at autism diagnosis (Loomes et al., 2017; van’t Hof et al., 2020; Zeidan et al., 2022); and (b) not included understudied autistic subpopulations, such as those without a formal diagnosis, or those with co-occurring intellectual, cognitive, or learning difficulties, or with greater daily support needs (Libsack et al., 2021; Petrolini et al., 2023). Therefore, more diverse and representative samples are needed to establish whether camouflaging generalises across the entire autism spectrum.

In addition to these suggestions for researchers, the findings from this review have implications for clinicians who work with autistic people. Clinicians are cautioned against the use of the CAT-Q as a screening tool because its clinical validity has not been firmly established, and its specificity to the experiences of autistic people is yet to be confirmed. To inform their clinical decision-making processes, clinicians are also urged to consider competing explanations for behaviours that clients may self-identify as camouflaging/masking, such as social anxiety (see Lau et al., 2019; Wood & Gadow, 2010, for discussions about the nature of anxiety in autistic people), self-presentation (Ai et al., 2022), concealment (see, for example, Pachankis, 2007) and social learning strategies (e.g. Bandura, 1977, 1986). However, more conceptual analytic research is needed to further increase clarity of camouflaging and to provide concrete guidance to clinicians as to how these constructs might be differentiated.

## Conclusion

The findings of this review support a need for more clarity and methodological rigour in camouflaging research. The conceptual issues identified by this review may inform future conceptual analyses to draw clearer boundaries between camouflaging and other behavioural concepts. In turn, this increased clarity may inform clinical interpretations of camouflaging and the further development of camouflaging measures, particularly by reducing sources of confounding and enhancing their construct validity.

## Supplemental Material

sj-docx-1-aut-10.1177_13623613261420085 – Supplemental material for Camouflaging and autism: Conceptualisation and methodological issues Supplemental material, sj-docx-1-aut-10.1177_13623613261420085 for Camouflaging and autism: Conceptualisation and methodological issues by Wayne M Arnold, Vicki Bitsika and Christopher F Sharpley in Autism

## Notes

1. Although some autistic people have reported a preference for the term ‘masking’ instead of ‘camouflaging’ (Hechler et al., 2025; Pearson & Rose, 2021), large-scale empirical studies have not been conducted on these term preferences with the autistic community, and arguments against both of these terms have also been presented (W. B. Lawson, 2020). As such, there is currently no consensus on which terms to use (Fombonne, 2020; Lai et al., 2020; Libsack et al., 2021). ‘Camouflaging’ was the preferred primary term in this article because it is the most widely used term in autism research.

2. The appropriateness of social skills training (SST) in ameliorating difficulties with social communication/interaction that may arise from autistic traits is sometimes questioned because these interventions may encourage inauthentic forms of social behaviour, such as camouflaging (Bottema-Beutel et al., 2018). However, more research is needed to determine whether the social behavioural changes that occur as a result of SST are equivalent to camouflaging.
