# Supplementary material for: Camouflaging and autism: Conceptualisation and methodological issues
Source: Autism. 2026 Feb 21;30(5):1131–46. doi: 10.1177/13623613261420085 (PMC13087159; doi:10.1177/13623613261420085)
Supplement: sj-docx-1-aut-10.1177_13623613261420085 – Supplemental material for Camouflaging and autism: Conceptualisation and methodological issues [file sj-docx-1-aut-10.1177_13623613261420085.docx]

**Supplemental Material**

**Figure S1**

*Flow Diagram*

**Identification of studies via other methods**

**Identification of studies via databases**

Records identified from:

Google Scholar
(n = 5917)

Records removed *before screening*:

Duplicate records removed
(n = 832)

Records identified from:

Backward citation searching of 193 articles retrieved from database searching
(n = 1039)

**Identification**

Articles included in review

(n = 389)

**Included**

Reports sought for retrieval

(n = 193)

Reports not retrieved

(n = 0)

Records screened

(n = 5917)

Records excluded (n = 5724):

Wrong concept (e.g., COVID-19 ‘masking’; ‘compensation’ for study participation)
(n = 4761)

Wrong record type (e.g., book chapters, news media)
(n = 846)

Not English language (n=117)

**Screening**

Reports sought for retrieval
(n = 196)

Reports excluded (n = 11):

Did not refer to camouflaging concept (n = 11)

Reports assessed for eligibility
(n = 207)

Reports not retrieved

(n = 0)

Source: Page MJ, et al. BMJ 2021;372:n71. doi: 10.1136/bmj.n71.

This work is licensed under CC BY 4.0. To view a copy of this license, visit <https://creativecommons.org/licenses/by/4.0/>

**Table S1**

*List of Camouflaging-Related Studies Reviewed and Their Variable Applications of Terminology*

| **Primary term used** | | **List of studies** | **Number of studies (% of total)** |
| --- | --- | --- | --- |
| **Studies which used multiple terms interchangeably to describe a single behavioural phenomenon** | | | **230 (59.1%)** |
|  | Adaptive morphing | Black et al. (2023); Lawson (2020) | 2 (0.5%) |
|  | Camouflaging | Adams et al. (2023); Allely (2019a); A. H. Anderson et al. (2020); Angulo et al. (2019); Atkinson et al. (2025); Backer van Ommeren et al. (2017); Bernardin, Mason, et al. (2021); Bhargava and Ashwin (2025); Bitsika et al. (2021); Blackhurst et al. (2025); Boorse et al. (2019); Bradley et al. (2021); Bradley et al. (2024); Brake (2024); Burroughs et al. (2024); Cage et al. (2018, 2019); Cage and Troxell-Whitman (2019, 2020); Carminati et al. (2024); Carpita et al. (2024); Cook et al. (2024); Cooper (2024); Crompton et al. (2020); Davies et al. (2024); Day et al. (2024); de la Roche and Kelley (2024); Dean et al. (2017); Dean and Nordahl-Hansen (2024); Dell'Osso et al. (2025); den Hartog et al. (2023); Durben (2024); Duvekot et al. (2017); Eckerd (2020); Ellestad et al. (2023); Evans et al. (2019); Field et al. (2024); Finn et al. (2023); Forster and Pearson (2020); Franklin et al. (2024); Giroux et al. (2024); Glanville et al. (2025); Gonçalves Garcia et al. (2025); Goscicki et al. (2025); Gosling et al. (2023); Gould (2017); Graf-Kurtulus and Gelo (2025); Green et al. (2019); Hake (2025); Harmens et al. (2022); Head et al. (2014); Hernández et al. (2024); Holingue et al. (2025); Hong et al. (2025); Huang et al. (2020); Hull and Mandy (2017); Jolliffe et al. (2025); Kanfiszer et al. (2017); Karaminis et al. (2024); Kentrou et al. (2021); Khudiakova et al. (2025); Khudiakova, Russell, et al. (2024); Kirkovski et al. (2013); Knutsen et al. (2019); Koteyko et al. (2024); Lai and Baron-Cohen (2015); Lai et al. (2015); Lai et al. (2011); Lai et al. (2017); Lai and Szatmari (2020); Lam et al. (2024); Leedham et al. (2020); Lei et al. (2025); Lewis (2017); Lewis and Stevens (2023); Lilley et al. (2022); Loomes et al. (2017); Lu et al. (2023); Lundin et al. (2020); Mandy and Tchanturia (2015); McQuaid et al. (2023); Mesa and Hamilton (2022); Milner, Colvert, et al. (2022); Milner et al. (2019); Miranda-Ojeda et al. (2025); Muggleton et al. (2019); Muratori et al. (2024); Myles et al. (2019); Nieradka and Kossewska (2023); Okamoto et al. (2024); Øverland et al. (2024); Pelton et al. (2020); Perry et al. (2021); Petrolini et al. (2023); Ratto et al. (2018); Ridgway et al. (2024); Riebel et al. (2024); Rippon (2024); Rivera and Bennetto (2023); Ross et al. (2022); Rynkiewicz et al. (2019); Schoondermark et al. (2024); Schuck et al. (2019); Sedgewick, Hill, et al. (2019); Simcoe et al. (2023); Somerville et al. (2023); Strang et al. (2020); Summerill and Summers (2025); Tafolla et al. (2025); Tien, Pearson, et al. (2025); Trunk et al. (2024); Tubío-Fungueiriño et al. (2020); van der Putten, Mol, et al. (2023); Walsh et al. (2023); Weiner et al. (2025); Wood-Downie et al. (2020); Yau et al. (2023); Young et al. (2018); Zakai-Mashiach (2023); Zhou and Kim (2024); Zhuang et al. (2024); Zhuang et al. (2023) | 122 (31.4%) |
|  | Compensation | Garvey et al. (2024); Jellett and Muggleton (2022); Jensen et al. (2024); Marocchini (2023); Parish-Morris (2019) | 5 (1.3%) |
|  | Concealment | Botha et al. (2020); Botha and Frost (2020); Botha and Gillespie-Lynch (2022); Davidson and Henderson (2010); Farsinejad et al. (2022); Frost et al. (2019); Hennekam et al. (2024); Khudiakova, Le Forestier, et al. (2024); Lindsay et al. (2019); Ng and Ng (2022); O'Connor et al. (2018); Thompson-Hodgetts et al. (2020); Underhill et al. (2019) | 13 (3.3%) |
|  | False self | Hickey et al. (2017) | 1 (0.3%) |
|  | Imitation | Attwood (2000) | 1 (0.3%) |
|  | Masking | Accardo et al. (2024); J. Anderson et al. (2020); Arnold et al. (2023a); Baldwin and Costley (2016); Banks et al. (2024); Benedetto (2024); Brett et al. (2024); Bury et al. (2022); Cage et al. (2022); Chapman et al. (2022); Cleary et al. (2023); Collins and Metcalfe (2024); Cook et al. (2018); Craddock (2024); Cridland et al. (2014); Dabbs et al. (2024); Diemer et al. (2022); Evans et al. (2023); Fletcher et al. (2024); Gemma (2021); Grant et al. (2024); Gurba et al. (2024); Hechler et al. (2025); Higgins et al. (2021); Hill (2024); Iacomini et al. (2024); Lai et al. (2023); Lai et al. (2022); Leadbitter et al. (2021); Leaf et al. (2023); Lei, Cooper, et al. (2024); Lei and Nocon (2024); Lilley et al. (2021); Long et al. (2024); Mantzalas, Richdale, Adikari, et al. (2022); Mantzalas, Richdale and Dissanayake (2022); McAuliffe et al. (2022); Miller et al. (2021); Milton and Sims (2016); Morrison et al. (2019); Murphy et al. (2022); Nagar Shimoni et al. (2025); Norvaišaite and Tateo (2024); Oliver et al. (2024); Ormond et al. (2018); Pearson and Rose (2021); Pearson et al. (2022); Pellicano and den Houting (2021); Pellicano et al. (2022); Pryke-Hobbes et al. (2023); Quigley et al. (2024); Raymaker et al. (2020); Rhodes et al. (2023); Robinson and Crane (2025); Sánchez-Pedroche et al. (2025); Schuck et al. (2024); Seers and Hogg (2022); Shaw et al. (2023); Smith and Jones (2020); Syharat et al. (2023); Tien, Wolpe, et al. (2025); Tierney et al. (2016); Tomlinson et al. (2020); Urbaniak and D’Amico (2024); Venkatesan and Tolani (2024); Venter et al. (2022); Zakai-Mashiach (2025); Zener (2019); Zolyomi et al. (2019) | 69 (17.7%) |
|  | Masquerading | Carrington and Graham (2001); Carrington et al. (2003); Cresswell et al. (2019); Humphrey and Lewis (2008); Jarman and Rayner (2015); Mogensen and Mason (2015) | 6 (1.5%) |
|  | Non-disclosure | Huang et al. (2022); O’Connor et al. (2020) | 2 (0.5%) |
|  | Passing | Bottema-Beutel et al. (2018); Cox et al. (2017); Libsack et al. (2021) | 3 (0.8%) |
|  | Pretending to be normal | Bargiela et al. (2016) | 1 (0.3%) |
|  | Social morphing | Kuzminski et al., 2024 | 1 (0.3%) |
|  | Suppression | Charlton et al. (2021); Collis et al. (2022); Johnson and Joshi (2016); Kapp et al. (2019) | 4 (1.0%) |
| **Studies which used separate terms to represent distinct categories of behaviour** | | | **74 (19.0%)** |
|  | Camouflaging | Ai et al. (2024); Allely (2019b); Attaullah et al. (2023); Beck et al. (2020); Calderoni (2022); Califano et al. (2024); Cassidy et al. (2023); Cassidy et al. (2019); Cook, Crane, Bourne, et al. (2021); Cook, Crane, Hull, et al. (2021); Corbett et al. (2020); Costache et al. (2024); Cruz et al. (2024); Dell'Osso et al. (2022); Dell’Osso et al. (2021); Galvin et al. (2024); Graham et al. (2023); Halsall et al. (2021); Han et al. (2022); Hannon et al. (2022); Hongo et al. (2024); Howe et al. (2023); Hull, Lai, et al. (2020); Hull et al. (2021); Hull et al. (2024); Hull et al. (2019); Hull et al. (2017); Hull, Petrides, et al. (2020); Jedrzejewska and Dewey (2022); Jorgenson et al. (2020); Keating et al. (2024); Klein et al. (2024); Klein and Macoun (2025); Kong et al. (2024); Lai et al. (2019); Lei, Leigh, et al. (2024b); Lei, Mason, et al. (2024); Loo et al. (2023); Lorenz and Hull (2024); Lynch et al. (2024); Mahony and O'Ryan (2022); McKinney et al. (2024); McKinnon et al. (2024); Milner et al. (2023); Milner, Mandy, et al. (2022); Moore et al. (2023); Mosquera et al. (2021); Mosquera et al. (2022); O'Loghlen and Lang (2023); Oshima et al. (2024); Pyszkowska (2024); Remnélius et al. (2024); Riebel et al. (2025); Robinson et al. (2020); Scheerer et al. (2020); Schneid and Raz (2020); Stroud et al. (2025); Tamura et al. (2024); van der Putten et al. (2024); van der Putten, van Rentergem, et al. (2023); Weiner et al. (2023); Williams (2022) | 62 (15.9%) |
|  | Compensation | Livingston, Colvert, et al. (2019); Livingston and Happé (2017); Livingston, Shah, et al. (2019); Livingston et al. (2020); Mazurek et al. (2024) | 5 (1.3%) |
|  | Concealment | Underhill et al. (2024) | 1 (0.3%) |
|  | Impression management | Ai et al. (2022, 2023); Khudiakova, Alexandrovsky, et al. (2024) | 3 (0.8%) |
|  | Masking | McMahon et al. (2020); Radulski (2022) | 2 (0.5%) |
|  | Social strategies | Funawatari, Sumiya, Iwabuchi and Senju (2024) | 1 (0.3%) |
| **Studies which used one term to represent a single behavioural phenomenon** | | | **74 (19.0%)** |
|  | Camouflaging | Benatov et al. (2025); Bitsika and Sharpley (2019, 2020, 2024); Cage and Burton (2019); Camm-Crosbie et al. (2019); Capiotto et al. (2024); Corbett et al. (2024); Dachez et al. (2024); Datu (2025); Davis and Crompton (2021); den Houting et al. (2021); Ferri et al. (2018); Funawatari, Sumiya, Iwabuchi, Nishimura, et al. (2024); Harrop et al. (2019); Harrop et al. (2018); Huang et al. (2023); Hughes et al. (2024); Johnson and Joshi (2014); Kaat et al. (2020); Koteyko et al. (2022); Kuo et al. (2024); Lawson (2019); Lawson et al. (2018); Lehnhardt et al. (2016); Mademtzi et al. (2018); Mandy and Lai (2017); McFayden et al. (2023); Moseley et al. (2018); Mussey et al. (2017); Parish-Morris et al. (2017); Porricelli et al. (2024); Richdale et al. (2025); Rynkiewicz et al. (2016); Sasson and Morrison (2019); Savard et al. (2024); Scheerer et al. (2024); Schiltz et al. (2024); Sedgewick, Crane, et al. (2019); Stark et al. (2024); Stockwell et al. (2020); Toda et al. (2024); Trundle et al. (2022); van Dijk et al. (2024); White et al. (2024); Wiskerke et al. (2018); Wood‐Downie et al. (2021); Zhang and Colizzi (2025) | 48 (12.3%) |
|  | Compensation | Fiebich (2017); Teunisse and De Gelder (2001) | 2 (0.5%) |
|  | Concealment | Sturrock et al. (2022) | 1 (0.3%) |
|  | Masking | Aiston et al. (2025); Beck et al. (2024); Billington et al. (2024); Chazin et al. (2024); Collis et al. (2024); Crompton et al. (2024); Ferguson et al. (2024); Foster et al. (2025); A. Grant et al. (2025); Husmann et al. (2024); Krishnan et al. (2024); McMorris et al. (2024); McMurtry et al. (2025); Morris et al. (2025); Overton et al. (2023); Phillips (2024); Shalit et al. (2024); Sullivan (2024); Tamilson et al. (2024) | 19 (4.9%) |
|  | Non-disclosure | Tomas et al. (2022); Treweek et al. (2019) | 2 (0.5%) |
|  | Passing | Milton and Lyte (2012); White et al. (2020) | 2 (0.5%) |
| **Studies which used the same terms both interchangeably and distinctly** | | | **11 (2.8%)** |
|  | Camouflaging | Alaghband-Rad et al. (2023); Arnold et al. (2023b); Belcher et al. (2021); Bernardin, Lewis, et al. (2021); Cook, Hull, et al. (2021); Cook et al. (2023); S. Grant et al. (2025); Lei, Leigh, et al. (2024a); Pérez-Arqueros et al. (2025); Wicherkiewicz and Gambin (2024) | 10 (2.6%) |
|  | Masking | Mantzalas et al. (2024) | 1 (0.3%) |

*Note.* Total number of studies = 389. ‘Primary’ terms were determined by using a combination of metrics: (a) frequency of use; (b) explicit statements by authors about their preferred term or system of categorisation of concepts; and/or (c) visibility and prioritisation of terms (e.g., use in title/abstract). Categories are sorted by the proportion of total studies within them. Primary terms are sorted alphabetically within each category. Some terms listed in Table 1 were not used as primary terms (i.e., they were used as synonyms or as sub-categories of primary terms) and thus do not appear here.

**References**

Accardo, A. L., Bomgardner, E. M., Rubinstein, M. B., & Woodruff, J. (2024). Valuing neurodiversity on campus: Perspectives and priorities of neurodivergent students, faculty, and professional staff. *Journal of Diversity in Higher Education*, No Pagination Specified-No Pagination Specified. <https://doi.org/10.1037/dhe0000571>

Adams, D., Ambrose, K., Simpson, K., Malone, S., & Dargue, N. (2023). The relationship between anxiety and social outcomes in autistic children and adolescents: A meta-analysis. *Clinical Child and Family Psychology Review*, *26*(3), 706-720. <https://doi.org/10.1007/s10567-023-00450-7>

Ai, W., Cunningham, W. A., & Lai, M.-C. (2022). Reconsidering autistic ‘camouflaging’ as transactional impression management. *Trends in Cognitive Sciences*, *26*(8), 631-645. <https://doi.org/10.1016/j.tics.2022.05.002>

Ai, W., Cunningham, W. A., & Lai, M.-C. (2023). The dimensional structure of the camouflaging autistic traits questionnaire (CAT-Q) and predictors of camouflaging in a representative general population sample. *Comprehensive Psychiatry*, 152434. <https://doi.org/10.1016/j.comppsych.2023.152434>

Ai, W., Cunningham, W. A., & Lai, M.-C. (2024). Camouflaging, internalized stigma, and mental health in the general population. *International Journal of Social Psychiatry*, *70*(7), 1239-1253. <https://doi.org/10.1177/00207640241260020>

Aiston, J., Koteyko, N., & van Driel, M. (2025). Discourse-based approaches to autistic focussed interests: Understanding shared focus, mutual accommodation, and multimodal expression. *Applied Linguistics*, amaf006. <https://doi.org/10.1093/applin/amaf006>

Alaghband-Rad, J., Hajikarim-Hamedani, A., & Motamed, M. (2023). Camouflage and masking behavior in adult autism. *Front Psychiatry*, *14*, 1108110. <https://doi.org/10.3389/fpsyt.2023.1108110>

Allely, C. S. (2019a). Exploring the female autism phenotype of repetitive behaviours and restricted interests (RBRIs): a systematic PRISMA review. *Advances in Autism*, *5*(3), 171-186. <https://doi.org/10.1108/aia-09-2018-0030>

Allely, C. S. (2019b). Understanding and recognising the female phenotype of autism spectrum disorder and the “camouflage” hypothesis: a systematic PRISMA review. *Advances in Autism*, *5*(1), 14-37. <https://doi.org/10.1108/AIA-09-2018-0036>

Anderson, A. H., Stephenson, J., & Carter, M. (2020). Perspectives of Former Students with ASD from Australia and New Zealand on Their University Experience. *Journal of Autism and Developmental Disorders*. <https://doi.org/10.1007/s10803-020-04386-7>

Anderson, J., Marley, C., Gillespie-Smith, K., Carter, L., & Macmahon, K. (2020). When the mask comes off: Mothers’ experiences of parenting a daughter with autism spectrum condition. *Autism*, 136236132091366. <https://doi.org/10.1177/1362361320913668>

Angulo, H., Chan, M., & DeThorne, L. (2019). Life Is a Stage: Autistic Perspectives on Neurotypicality. *Autism Adulthood*, *1*(4), 276-285. <https://doi.org/10.1089/aut.2019.0024>

Arnold, S. R. C., Higgins, J. M., Weise, J., Desai, A., Pellicano, E., & Trollor, J. N. (2023a). Confirming the nature of autistic burnout. *Autism*, 13623613221147410. <https://doi.org/10.1177/13623613221147410>

Arnold, S. R. C., Higgins, J. M., Weise, J., Desai, A., Pellicano, E., & Trollor, J. N. (2023b). Towards the measurement of autistic burnout. *Autism*, 13623613221147401. <https://doi.org/10.1177/13623613221147401>

Atkinson, E., Wright, S., & Wood-Downie, H. (2025). “Do My Friends Only Like the School Me or the True Me?”: School Belonging, Camouflaging, and Anxiety in Autistic Students. *Journal of Autism and Developmental Disorders*. <https://doi.org/10.1007/s10803-024-06668-w>

Attaullah, S., Khalil, S., & Qamar, R. F. (2023). Mediating Role of Perceived Stigma and Camouflaging in Relationship between Autistic Identity and Wellbeing among Autistic Adults. *Journal of Development and Social Sciences*, *4*(3), 821-831. <https://doi.org/10.47205/jdss.2023(4-III)75>

Attwood, T. (2000). Strategies for Improving the Social Integration of Children with Asperger Syndrome. *Autism*, *4*(1), 85-100. <https://doi.org/10.1177/1362361300004001006>

Backer van Ommeren, T., Koot, H. M., Scheeren, A. M., & Begeer, S. (2017). Sex differences in the reciprocal behaviour of children with autism. *Autism*, *21*(6), 795-803. <https://doi.org/10.1177/1362361316669622>

Baldwin, S., & Costley, D. (2016). The experiences and needs of female adults with high-functioning autism spectrum disorder. *Autism*, *20*(4), 483-495. <https://doi.org/10.1177/1362361315590805>

Banks, A. L., Mainess, K. J., Javaherian, H., & Natsuaki, M. N. (2024). “Very Misunderstood”: Self-Perceived Social Communication Experiences of Autistic Young Adults. *Youth*, *4*(4), 1628-1646.

Bargiela, S., Steward, R., & Mandy, W. (2016). The Experiences of Late-diagnosed Women with Autism Spectrum Conditions: An Investigation of the Female Autism Phenotype. *Journal of Autism and Developmental Disorders*, *46*(10), 3281-3294. <https://doi.org/10.1007/s10803-016-2872-8>

Beck, J. S., Lundwall, R. A., Gabrielsen, T., Cox, J. C., & South, M. (2020). Looking good but feeling bad: “Camouflaging” behaviors and mental health in women with autistic traits. *Autism*, *24*(4), 809-821. <https://doi.org/10.1177/1362361320912147>

Beck, K. B., MacKenzie, K. T., Kumar, T., Breitenfeldt, K. E., Chang, J. C., Conner, C. M., Mandell, D. L., White, S. W., & Mazefsky, C. A. (2024). “The World’s Really Not Set Up for the Neurodivergent Person”: Understanding Emotion Dysregulation from the Perspective of Autistic Adults. *Autism in Adulthood*. <https://doi.org/10.1089/aut.2023.0214>

Belcher, H. L., Morein-Zamir, S., Mandy, W., & Ford, R. M. (2021). Camouflaging Intent, First Impressions, and Age of ASC Diagnosis in Autistic Men and Women. *Journal of Autism and Developmental Disorders*. <https://doi.org/10.1007/s10803-021-05221-3>

Benatov, J., Sarel-Mahlev, E., & Bar Yehuda, S. (2025). Camouflage, Burnout-Exhaustion, and Depression in Autistic Adults. *Autism in Adulthood*. <https://doi.org/10.1089/aut.2024.0147>

Benedetto, M. S. (2024). The “Dual Masking Phenomenon”: A Critical Autoethnography of an Autistic Multiracial Latina Amid the Presence of White Autistic Researchers and Self-Advocates. *Autism in Adulthood*. <https://doi.org/10.1089/aut.2024.0105>

Bernardin, C. J., Lewis, T., Bell, D., & Kanne, S. (2021). Associations between social camouflaging and internalizing symptoms in autistic and non-autistic adolescents. *Autism*, 136236132199728. <https://doi.org/10.1177/1362361321997284>

Bernardin, C. J., Mason, E., Lewis, T., & Kanne, S. (2021). “You Must Become a Chameleon to Survive”: Adolescent Experiences of Camouflaging. *Journal of Autism and Developmental Disorders*, *51*(12), 4422-4435. <https://doi.org/10.1007/s10803-021-04912-1>

Bhargava, A., & Ashwin, C. (2025). Barriers and facilitators towards an autism diagnosis for females within healthcare: A thematic analysis of interviews with UK healthcare professionals. *Research in Autism*, *121-122*, 202547. <https://doi.org/10.1016/j.reia.2025.202547>

Billington, J., Loucas, T., & Knott, F. (2024). “I liked school, but school didn’t like me”: Autistic young adults’ reflections on their mainstream primary school experiences. *Neurodiversity*, *2*, 27546330241310174. <https://doi.org/10.1177/27546330241310174>

Bitsika, V., & Sharpley, C. F. (2019). Effects of Diagnostic Severity upon Sex Differences in Behavioural Profiles of Young Males and Females with Autism Spectrum Disorder. *Journal of Autism and Developmental Disorders*, *49*(11), 4429-4440. <https://doi.org/10.1007/s10803-019-04159-x>

Bitsika, V., & Sharpley, C. F. (2020). Self- vs Parent Reports of Generalised Anxiety Disorder Symptomatology in Mildly Impaired Girls with an Autism Spectrum Disorder. *Journal of Autism and Developmental Disorders*, *50*(3), 1045-1055. <https://doi.org/10.1007/s10803-019-04339-9>

Bitsika, V., & Sharpley, C. F. (2024). The Association Between IQ and Its Components and Anxiety in Autistic Girls. *Advances in Neurodevelopmental Disorders*. <https://doi.org/10.1007/s41252-024-00415-z>

Bitsika, V., Sharpley, C. F., Mandy, W., McMillan, M. E., & Agnew, L. L. (2021). Girls’ cortisol concentrations, mothers’ anxiety, and self- versus parent-ratings of autistic girls’ anxiety. *Research in Autism Spectrum Disorders*, *81*, 101718. <https://doi.org/10.1016/j.rasd.2020.101718>

Black, M. H., Clarke, P. J. F., Deane, E., Smith, D., Wiltshire, G., Yates, E., Lawson, W. B., & Chen, N. T. M. (2023). “That impending dread sort of feeling”: Experiences of social interaction from the perspectives of autistic adults. *Research in Autism Spectrum Disorders*, *101*, 102090. <https://doi.org/10.1016/j.rasd.2022.102090>

Blackhurst, T., Warmelink, L., Roestorf, A., & Hartley, C. (2025). Exploring lie frequency and emotional experiences of deceptive decision-making in autistic adults. *Autism*, 13623613251315892. <https://doi.org/10.1177/13623613251315892>

Boorse, J., Cola, M., Plate, S., Yankowitz, L., Pandey, J., Schultz, R. T., & Parish-Morris, J. (2019). Linguistic markers of autism in girls: evidence of a “blended phenotype” during storytelling. *Molecular Autism*, *10*(1). <https://doi.org/10.1186/s13229-019-0268-2>

Botha, M., Dibb, B., & Frost, D. M. (2020). "Autism is me": an investigation of how autistic individuals make sense of autism and stigma. *Disability & Society*, 1-27. <https://doi.org/10.1080/09687599.2020.1822782>

Botha, M., & Frost, D. M. (2020). Extending the Minority Stress Model to Understand Mental Health Problems Experienced by the Autistic Population. *Society and Mental Health*, *10*(1), 20-34. <https://doi.org/10.1177/2156869318804297>

Botha, M., & Gillespie-Lynch, K. (2022). Come as You Are: Examining Autistic Identity Development and the Neurodiversity Movement through an Intersectional Lens. *Human Development*, *66*(2), 93-112. <https://doi.org/10.1159/000524123>

Bottema-Beutel, K., Park, H., & Kim, S. Y. (2018). Commentary on Social Skills Training Curricula for Individuals with ASD: Social Interaction, Authenticity, and Stigma. *Journal of Autism and Developmental Disorders*, *48*(3), 953-964. <https://doi.org/10.1007/s10803-017-3400-1>

Bradley, L., Shaw, R., Baron-Cohen, S., & Cassidy, S. (2021). Autistic Adults' Experiences of Camouflaging and Its Perceived Impact on Mental Health. *Autism in Adulthood*. <https://doi.org/10.1089/aut.2020.0071>

Bradley, S., Moore, F., Duffy, F., Clark, L., Suratwala, T., Knightsmith, P., & Gillespie-Smith, K. (2024). Camouflaging, not sensory processing or Autistic identity, predicts eating disorder symptoms in Autistic adults. *Autism*. <https://doi.org/10.1177/13623613241245749>

Brake, J. (2024). Between gaining acceptance and avoiding harm: navigating stigma and its consequences among autistic individuals. *Disability & Society*, 1-26. <https://doi.org/10.1080/09687599.2024.2374491>

Brett, S. G., den Houting, J. E., Black, M. H., Lawson, L. P., Trollor, J., & Arnold, S. R. C. (2024). Suitability of the DSM-5 social anxiety disorder severity scale for autistic adults. *Autism*, 13623613241290547. <https://doi.org/10.1177/13623613241290547>

Burroughs, C., Muscatello, R. A., & Corbett, B. A. (2024). The Role of Everyday Executive Function in Observed Social Symptoms of Autism Spectrum Disorder. *Journal of Autism and Developmental Disorders*. <https://doi.org/10.1007/s10803-024-06351-0>

Bury, S. M., Haschek, A., Wenzel, M., Spoor, J. R., & Hedley, D. (2022). Brief Report: Learning About Autism: Is the Source of Autism Knowledge Associated with Differences in Autism Knowledge, Autism Identity, and Experiences of Stigma. *Journal of Autism and Developmental Disorders*. <https://doi.org/10.1007/s10803-022-05823-5>

Cage, E., & Burton, H. (2019). Gender Differences in the First Impressions of Autistic Adults. *Autism Research*, *12*(10), 1495-1504. <https://doi.org/10.1002/aur.2191>

Cage, E., Cranney, R., & Botha, M. (2022). Brief Report: Does Autistic Community Connectedness Moderate the Relationship Between Masking and Wellbeing? *Autism in Adulthood*. <https://doi.org/10.1089/aut.2021.0096>

Cage, E., Di Monaco, J., & Newell, V. (2018). Experiences of Autism Acceptance and Mental Health in Autistic Adults. *Journal of Autism and Developmental Disorders*, *48*(2), 473-484. <https://doi.org/10.1007/s10803-017-3342-7>

Cage, E., Di Monaco, J., & Newell, V. (2019). Understanding, attitudes and dehumanisation towards autistic people. *Autism*, *23*(6), 1373-1383. <https://doi.org/10.1177/1362361318811290>

Cage, E., & Troxell-Whitman, Z. (2019). Understanding the Reasons, Contexts and Costs of Camouflaging for Autistic Adults. *Journal of Autism and Developmental Disorders*, *49*(5), 1899-1911. <https://doi.org/10.1007/s10803-018-03878-x>

Cage, E., & Troxell-Whitman, Z. (2020). Understanding the Relationships Between Autistic Identity, Disclosure, and Camouflaging. *Autism in Adulthood*. <https://doi.org/10.1089/aut.2020.0016>

Calderoni, S. (2022). Sex/gender differences in children with autism spectrum disorder: A brief overview on epidemiology, symptom profile, and neuroanatomy. *Journal of Neuroscience Research*, *n/a*(n/a). <https://doi.org/10.1002/jnr.25000>

Califano, M., Pruccoli, J., Martucci, M., Visconti, C., Barasciutti, E., Sogos, C., & Parmeggiani, A. (2024). Autism Spectrum Disorder Traits Predict Interoceptive Deficits and Eating Disorder Symptomatology in Children and Adolescents with Anorexia Nervosa—A Cross-Sectional Analysis: Italian Preliminary Data. *Pediatric Reports*, *16*(4), 1077-1088.

Camm-Crosbie, L., Bradley, L., Shaw, R., Baron-Cohen, S., & Cassidy, S. (2019). ‘People like me don’t get support’: Autistic adults’ experiences of support and treatment for mental health difficulties, self-injury and suicidality. *Autism*, *23*(6), 1431-1441. <https://doi.org/10.1177/1362361318816053>

Capiotto, F., Romano Cappi, G., Mirlisenna, I., Mazza, A., Cicinelli, G., Lauritano, C., Keller, R., & Dal Monte, O. (2024). Autonomic and hedonic response to affective touch in autism spectrum disorder. *Autism Research*, *17*(5), 923-933. <https://doi.org/10.1002/aur.3143>

Carminati, G. G., Zecca, G., & Carminati, F. (2024). Autism Spectrum Disorders and Borderline Personality Disorders: Comorbidity and Difficulty of Diagnosis in Women. *Psychology*, *15*(10), 1595-1613. <https://doi.org/10.4236/psych.2024.1510093>

Carpita, B., Nardi, B., Tognini, V., Poli, F., Amatori, G., Cremone, I. M., Pini, S., & Dell’Osso, L. (2024). Autistic Traits and Somatic Symptom Disorders: What Is the Link? *Brain Sciences*, *14*(3).

Carrington, S., & Graham, L. (2001). Perceptions of School by Two Teenage Boys with Asperger Syndrome and their Mothers: A Qualitative Study. *Autism*, *5*(1), 37-48. <https://doi.org/10.1177/1362361301005001004>

Carrington, S., Templeton, E., & Papinczak, T. (2003). Adolescents with Asperger Syndrome and Perceptions of Friendship. *Focus on Autism and Other Developmental Disabilities*, *18*(4), 211-218. <https://doi.org/10.1177/10883576030180040201>

Cassidy, S., McLaughlin, E., McGranaghan, R., Pelton, M., O'Connor, R., & Rodgers, J. (2023). Is camouflaging autistic traits associated with defeat, entrapment, and lifetime suicidal thoughts? Expanding the Integrated Motivational Volitional Model of Suicide. *Suicide and Life-Threatening Behavior*, *53*(4), 572-585. <https://doi.org/10.1111/sltb.12965>

Cassidy, S. A., Gould, K., Townsend, E., Pelton, M., Robertson, A. E., & Rodgers, J. (2019). Is Camouflaging Autistic Traits Associated with Suicidal Thoughts and Behaviours? Expanding the Interpersonal Psychological Theory of Suicide in an Undergraduate Student Sample. *Journal of Autism and Developmental Disorders*. <https://doi.org/10.1007/s10803-019-04323-3>

Chapman, L., Rose, K., Hull, L., & Mandy, W. (2022). “I want to fit in… but I don’t want to change myself fundamentally”: A qualitative exploration of the relationship between masking and mental health for autistic teenagers. *Research in Autism Spectrum Disorders*, *99*, 102069. <https://doi.org/10.1016/j.rasd.2022.102069>

Charlton, R. A., Entecott, T., Belova, E., & Nwaordu, G. (2021). “It feels like holding back something you need to say”: Autistic and Non-Autistic Adults accounts of sensory experiences and stimming. *Research in Autism Spectrum Disorders*, *89*, 101864. <https://doi.org/10.1016/j.rasd.2021.101864>

Chazin, K. T., Ledford, J. R., Wilson-Moses, J. M., Rajaraman, A., & Juárez, A. P. (2024). Centering Autistic Perspectives: Social Acceptability of Goals, Learning Contexts, and Procedures for Young Autistic Children. *Journal of Autism and Developmental Disorders*. <https://doi.org/10.1007/s10803-024-06242-4>

Cleary, M., West, S., Kornhaber, R., & Hungerford, C. (2023). Autism, Discrimination and Masking: Disrupting a Recipe for Trauma. *Issues in Mental Health Nursing*, *44*(9), 799-808. <https://doi.org/10.1080/01612840.2023.2239916>

Collins, J., & Metcalfe, D. (2024). Mental health, wellbeing and coping strategies of autistic adults during isolation. *Advances in Autism*, *10*(4), 367-381. <https://doi.org/10.1108/AIA-01-2024-0006>

Collis, E., Dark, E., Russell, A., & Brosnan, M. (2024). Self-Report of Restricted Repetitive Behaviors in Autistic Adults: A Systematic Review. *Autism in Adulthood*. <https://doi.org/10.1089/aut.2023.0111>

Collis, E., Gavin, J., Russell, A., & Brosnan, M. (2022). Autistic adults’ experience of restricted repetitive behaviours. *Research in Autism Spectrum Disorders*, *90*. <https://doi.org/10.1016/j.rasd.2021.101895>

Cook, A., Ogden, J., & Winstone, N. (2018). Friendship motivations, challenges and the role of masking for girls with autism in contrasting school settings. *European Journal of Special Needs Education*, *33*(3), 302-315. <https://doi.org/10.1080/08856257.2017.1312797>

Cook, J., Crane, L., Bourne, L., Hull, L., & Mandy, W. (2021). Camouflaging in an everyday social context: An interpersonal recall study. *Autism*, 136236132199264. <https://doi.org/10.1177/1362361321992641>

Cook, J., Crane, L., Hull, L., Bourne, L., & Mandy, W. (2021). Self-reported camouflaging behaviours used by autistic adults during everyday social interactions. *Autism*, 13623613211026754. <https://doi.org/10.1177/13623613211026754>

Cook, J., Hull, L., Crane, L., & Mandy, W. (2021). Camouflaging in autism: A systematic review. *Clinical Psychology Review*, *89*, 102080. <https://doi.org/10.1016/j.cpr.2021.102080>

Cook, J., Hull, L., & Mandy, W. (2024). Improving Diagnostic Procedures in Autism for Girls and Women: A Narrative Review. *Neuropsychiatric Disease and Treatment*, *20*(null), 505-514. <https://doi.org/10.2147/NDT.S372723>

Cook, J. M., Crane, L., & Mandy, W. (2023). Dropping the mask: It takes two. *Autism*, 13623613231183059. <https://doi.org/10.1177/13623613231183059>

Cooper, J. (2024). Autism, gender, and identity in college students. *Discover Psychology*, *4*(1), 26. <https://doi.org/10.1007/s44202-024-00116-7>

Corbett, B. A., Muscatello, R. A., Cyperski, M., Sadikova, E., Edmiston, E. K., McGonigle, T. W., Calvosa, R., & Vandekar, S. (2024). Gender diversity in autistic and neurotypical youth over adolescence and puberty: A longitudinal study. *Autism Research*, *17*(7), 1450-1463. <https://doi.org/10.1002/aur.3141>

Corbett, B. A., Schwartzman, J. M., Libsack, E. J., Muscatello, R. A., Lerner, M. D., Simmons, G. L., & White, S. W. (2020). Camouflaging in Autism: Examining Sex‐Based and Compensatory Models in Social Cognition and Communication. *Autism Research*. <https://doi.org/10.1002/aur.2440>

Costache, M. E., Gioia, F., Vanello, N., Greco, A., Lefebvre, F., Capobianco, A., Weibel, S., & Weiner, L. (2024). Exploring Emotion Control and Alexithymia in Autistic Adults: An Ecological Momentary Assessment Study. *Journal of Autism and Developmental Disorders*. <https://doi.org/10.1007/s10803-024-06551-8>

Cox, B. E., Thompson, K., Anderson, A., Mintz, A., Locks, T., Morgan, L., Edelstein, J., & Wolz, A. (2017). College Experiences for Students With Autism Spectrum Disorder: Personal Identity, Public Disclosure, and Institutional Support. *Journal of College Student Development*, *58*(1), 71-87. <https://doi.org/10.1353/csd.2017.0004>

Craddock, E. (2024). Being a Woman Is 100% Significant to My Experiences of Attention Deficit Hyperactivity Disorder and Autism: Exploring the Gendered Implications of an Adulthood Combined Autism and Attention Deficit Hyperactivity Disorder Diagnosis. *Qualitative Health Research*, *34*(14), 1442-1455. <https://doi.org/10.1177/10497323241253412>

Cresswell, L., Hinch, R., & Cage, E. (2019). The experiences of peer relationships amongst autistic adolescents: A systematic review of the qualitative evidence. *Research in Autism Spectrum Disorders*, *61*, 45-60. <https://doi.org/10.1016/j.rasd.2019.01.003>

Cridland, E. K., Jones, S. C., Caputi, P., & Magee, C. A. (2014). Being a Girl in a Boys’ World: Investigating the Experiences of Girls with Autism Spectrum Disorders During Adolescence. *Journal of Autism and Developmental Disorders*, *44*(6), 1261-1274. <https://doi.org/10.1007/s10803-013-1985-6>

Crompton, C. J., Fotheringham, F., Cebula, K., Webber, C., Foley, S., & Fletcher-Watson, S. (2024). Neurodivergent-designed and neurodivergent-led peer support in school: A feasibility and acceptability study of the neurodivergent peer support toolkit (NEST). *Neurodiversity*, *2*, 27546330241275248. <https://doi.org/10.1177/27546330241275248>

Crompton, C. J., Hallett, S., Ropar, D., Flynn, E., & Fletcher-Watson, S. (2020). ‘I never realised everybody felt as happy as I do when I am around autistic people’: A thematic analysis of autistic adults’ relationships with autistic and neurotypical friends and family. *Autism*, *24*(6), 1438-1448. <https://doi.org/10.1177/1362361320908976>

Cruz, S., Zubizarreta, S. C.-P., Costa, A. D., Araújo, R., Martinho, J., Tubío-Fungueiriño, M., Sampaio, A., Cruz, R., Carracedo, A., & Fernández-Prieto, M. (2024). Is There a Bias Towards Males in the Diagnosis of Autism? A Systematic Review and Meta-Analysis. *Neuropsychology Review*. <https://doi.org/10.1007/s11065-023-09630-2>

Dabbs, C. R., Hutchins, C. H., Baird, R., Scaer, A. J., Kosanovich, S. E., & Spitler-Nigh, B. (2024). Unmasking Bias: Autistic Perspectives in Mental Health Training. *Autism in Adulthood*. <https://doi.org/10.1089/aut.2024.0210>

Dachez, J., Seksek, S., Ete, N., Bianciotto, M., Toubhans, M.-P., Rachedi Nasri, Z., Bureau, R., & Garnier, P. (2024). Experiences of autistic people in supported employment in France: a participatory research project. *Advances in Autism*, *10*(4), 323-339. <https://doi.org/10.1108/AIA-03-2024-0022>

Datu, J. A. D. (2025). Is gratitude science inclusive? A scoping review on gratitude in individuals with diverse needs and exceptionalities. *Asian Journal of Social Psychology*, *28*(1), e12668. <https://doi.org/10.1111/ajsp.12668>

Davidson, J., & Henderson, V. L. (2010). ‘Coming out’ on the spectrum: autism, identity and disclosure. *Social & Cultural Geography*, *11*(2), 155-170. <https://doi.org/10.1080/14649360903525240>

Davies, J., Cooper, K., Killick, E., Sam, E., Healy, M., Thompson, G., Mandy, W., Redmayne, B., & Crane, L. (2024). Autistic identity: A systematic review of quantitative research. *Autism Research*, *n/a*(n/a). <https://doi.org/10.1002/aur.3105>

Davis, R., & Crompton, C. J. (2021). What Do New Findings About Social Interaction in Autistic Adults Mean for Neurodevelopmental Research? *Perspectives on Psychological Science*, *16*(3), 649-653. <https://doi.org/10.1177/1745691620958010>

Day, R., Simmons, L., Shade, E., Jennison, J., Allely, C. S., & Mukherjee, R. A. S. (2024). Exploring the presence of a sex-specific phenotype of autism spectrum disorder in a random cohort of males and females: a retrospective case note service audit. *Advances in Autism*, *ahead-of-print*(ahead-of-print). <https://doi.org/10.1108/AIA-11-2022-0053>

de la Roche, L., & Kelley, E. (2024). A Scoping Review of Sex/Gender Differences in Social Communication Skills and Behaviors of Autistic Youth—Are Sex/Gender-Specific Interventions Needed? *Review Journal of Autism and Developmental Disorders*. <https://doi.org/10.1007/s40489-024-00451-4>

Dean, M., Harwood, R., & Kasari, C. (2017). The art of camouflage: Gender differences in the social behaviors of girls and boys with autism spectrum disorder. *Autism*, *21*(6), 678-689. <https://doi.org/10.1177/1362361316671845>

Dean, M., & Nordahl-Hansen, A. (2024). The to be, or not to be, of acting autistic. *Autism*, 13623613241290270. <https://doi.org/10.1177/13623613241290270>

Dell'Osso, L., Carpita, B., Lorenzi, P., Toschi, D., & Amatori, G. (2025). The Brilliant Diva: clues of autism spectrum and mental disorder in the life of Hedy Lamarr. *Journal of Psychopathology*, *31*(1). <https://doi.org/10.36148/2284-0249-N643>

Dell'Osso, L., Cremone, I. M., Muti, D., Massimetti, G., Lorenzi, P., Carmassi, C., & Carpita, B. (2022). Validation of the Italian version of the Camouflaging Autistic Traits Questionnaire (CAT-Q) in a University population. *Comprehensive Psychiatry*, *114*, 152295. <https://doi.org/10.1016/j.comppsych.2022.152295>

Dell’Osso, L., Cremone, I. M., Chiarantini, I., Arone, A., Massimetti, G., Carmassi, C., & Carpita, B. (2021). Autistic traits and camouflaging behaviours: a cross-sectional investigation in a University student population. *CNS Spectrums*, 1-21. <https://doi.org/10.1017/S1092852921000808>

den Hartog, A., de la Roche, L., Derby, B., Psaradellis, E., & Kelley, E. (2023). A scoping review of sex/gender differences in pragmatic language and friendship characteristics in autistic children and adolescents. *Research in Autism Spectrum Disorders*, *108*, 102229. <https://doi.org/10.1016/j.rasd.2023.102229>

den Houting, J., Botha, M., Cage, E., Jones, D. R., & Kim, S. Y. (2021). Shifting stigma about autistic young people. *The Lancet Child & Adolescent Health*, *5*(12), 839-841. <https://doi.org/10.1016/S2352-4642(21)00309-6>

Diemer, M. C., Gerstein, E. D., & Regester, A. (2022). Autism presentation in female and Black populations: Examining the roles of identity, theory, and systemic inequalities. *Autism*, 13623613221113501. <https://doi.org/10.1177/13623613221113501>

Durben, D. L. (2024). Understanding autistic camouflaging: The use of online community discussions and stigmatized identity research. *Neurodiversity*, *2*, 27546330241266726. <https://doi.org/10.1177/27546330241266726>

Duvekot, J., van der Ende, J., Verhulst, F. C., Slappendel, G., van Daalen, E., Maras, A., & Greaves-Lord, K. (2017). Factors influencing the probability of a diagnosis of autism spectrum disorder in girls versus boys. *Autism*, *21*(6), 646-658. <https://doi.org/10.1177/1362361316672178>

Eckerd, M. (2020). Detection and Diagnosis of ASD in Females. *Journal of Health Service Psychology*, *46*(1), 37-47. <https://doi.org/10.1007/s42843-020-00006-1>

Ellestad, A. I., Beymer, L. L., & Villegas, S. (2023). The lived experiences of individuals with high-functioning autism during the job interview process: A phenomenological study. *Journal of Employment Counseling*, *n/a*(n/a). <https://doi.org/10.1002/joec.12212>

Evans, J. A., Krumrei-Mancuso, E. J., & Rouse, S. V. (2023). What You Are Hiding Could Be Hurting You: Autistic Masking in Relation to Mental Health, Interpersonal Trauma, Authenticity, and Self-Esteem. *Autism in Adulthood*. <https://doi.org/10.1089/aut.2022.0115>

Evans, S. C., Boan, A. D., Bradley, C., & Carpenter, L. A. (2019). Sex/Gender Differences in Screening for Autism Spectrum Disorder: Implications for Evidence-Based Assessment. *Journal of Clinical Child & Adolescent Psychology*, *48*(6), 840-854. <https://doi.org/10.1080/15374416.2018.1437734>

Farsinejad, A., Russell, A., & Butler, C. (2022). Autism disclosure – The decisions autistic adults make. *Research in Autism Spectrum Disorders*, *93*, 101936. <https://doi.org/10.1016/j.rasd.2022.101936>

Ferguson, A., Martin, D., & Pearson, A. (2024). “It Has Shown Me How Much I Am Capable Of”: An Exploration of Autistic Burnout Experiences in Motherhood. *Autism in Adulthood*. <https://doi.org/10.1089/aut.2024.0282>

Ferri, S. L., Abel, T., & Brodkin, E. S. (2018). Sex Differences in Autism Spectrum Disorder: a Review. *Current Psychiatry Reports*, *20*(2). <https://doi.org/10.1007/s11920-018-0874-2>

Fiebich, A. (2017). Pluralism, social cognition, and interaction in autism. *Philosophical Psychology*, *30*(1-2), 161-184. <https://doi.org/10.1080/09515089.2016.1261394>

Field, S. L., Williams, M. O., Jones, C. R. G., & Fox, J. R. E. (2024). A meta-ethnography of autistic people’s experiences of social camouflaging and its relationship with mental health. *Autism*, 13623613231223036. <https://doi.org/10.1177/13623613231223036>

Finn, M., Flower, R. L., Leong, H. M., & Hedley, D. (2023). ‘If I’m just me, I doubt I’d get the job’: A qualitative exploration of autistic people’s experiences in job interviews. *Autism*, 13623613231153480. <https://doi.org/10.1177/13623613231153480>

Fletcher, L., Rabagliati, H., & Culbertson, J. (2024). Autistic Traits, Communicative Efficiency, and Social Biases Shape Language Learning in Autistic and Allistic Learners. *Cognitive Science*, *48*(11), e70007. <https://doi.org/10.1111/cogs.70007>

Forster, S., & Pearson, A. (2020). “Bullies tend to be obvious”: autistic adults perceptions of friendship and the concept of ‘mate crime’. *Disability & Society*, *35*(7), 1103-1123. <https://doi.org/10.1080/09687599.2019.1680347>

Foster, S. J., Ackerman, R. A., Wilks, C. E. H., Dodd, M., Calderon, R., Ropar, D., Fletcher-Watson, S., Crompton, C. J., & Sasson, N. J. (2025). Rapport in same and mixed neurotype groups of autistic and non-autistic adults. *Autism*, 13623613251320444. <https://doi.org/10.1177/13623613251320444>

Franklin, M. D., Taylor, E. E., Floríndez, D. C., Guzman, M., Lawson, T. L., Rios, J., & Angell, A. M. (2024). An Occupational Science Contribution to Camouflaging Scholarship: Centering Intersectional Experiences of Occupational Disruptions. *Autism in Adulthood*. <https://doi.org/10.1089/aut.2023.0070>

Frost, K. M., Bailey, K. M., & Ingersoll, B. R. (2019). “I Just Want Them to See Me As…Me”: Identity, Community, and Disclosure Practices Among College Students on the Autism Spectrum. *Autism in Adulthood*, *1*(4), 268-275. <https://doi.org/10.1089/aut.2018.0057>

Funawatari, R., Sumiya, M., Iwabuchi, T., Nishimura, T., Komeda, H., & Senju, A. (2024). Camouflaging in Autistic Adults is Modulated by Autistic and Neurotypical Characteristics of Interaction Partners. *Journal of Autism and Developmental Disorders*. <https://doi.org/10.1007/s10803-024-06481-5>

Funawatari, R., Sumiya, M., Iwabuchi, T., & Senju, A. (2024). Double-Edged Effects of Social Strategies on the Well-Being of Autistic People: Impact of Self-Perceived Effort and Efficacy. *Brain Sciences*, *14*(10).

Galvin, J., Aguolu, P., Amos, A., Bayne, F., Hamza, F., & Alcock, L. (2024). Self-Compassion, Camouflaging, and Mental Health in Autistic Adults. *Autism in Adulthood*. <https://doi.org/10.1089/aut.2023.0110>

Garvey, A., Ryan, C., & Murphy, M. (2024). Deliberate and Self-Conscious Adaptation of Eye-Contact by Autistic Adults. *Journal of Autism and Developmental Disorders*. <https://doi.org/10.1007/s10803-024-06296-4>

Gemma, N. (2021). Reconceptualising ‘reasonable adjustments’ for the successful employment of autistic women. *Disability & Society*, 1-19. <https://doi.org/10.1080/09687599.2021.1971065>

Giroux, M., Courcy, I., & Nadig, A. (2024). Social Camouflage in Autism: An Analysis of Decision Making. *Autism in Adulthood*. <https://doi.org/10.1089/aut.2023.0077>

Glanville, B., Oates, J., Foley, K.-R., Hurem, A., Osmetti, L., & Allen, K. (2025). Harmonizing Identities: A Scoping Review on Voice and Communication Supports and Challenges for Autistic Trans and Gender Diverse Individuals. *Journal of Autism and Developmental Disorders*. <https://doi.org/10.1007/s10803-025-06768-1>

Gonçalves Garcia, S., da Silveira Simões-Pires, C., Acosta Brum, J., & Centurion Cabral, J. (2025). Taking Off the Mask: Investigating Autism Diagnosis and Camouflaging in Adult Women. *Autism in Adulthood*. <https://doi.org/10.1089/aut.2024.0150>

Goscicki, B. L., Scoggins, M. E., Espinosa, G. H., & Hodapp, R. M. (2025). A “Round, Bruising Sort of Pain”: Autistic Girls’ Social Camouflaging in Inclusive High School Settings. *Journal of Autism and Developmental Disorders*. <https://doi.org/10.1007/s10803-024-06716-5>

Gosling, J., Purrington, J., & Hartley, G. (2023). Exploring the Lived Experiences of Autistic Women: A Thematic Synthesis. *Review Journal of Autism and Developmental Disorders*. <https://doi.org/10.1007/s40489-023-00367-5>

Gould, J. (2017). Towards understanding the under-recognition of girls and women on the autism spectrum. *Autism*, *21*(6), 703-705. <https://doi.org/10.1177/1362361317706174>

Graf-Kurtulus, S., & Gelo, O. C. G. (2025). Rethinking psychological interventions in autism: Toward a neurodiversity-affirming approach. *Counselling and Psychotherapy Research*, *25*(1), e12874. <https://doi.org/10.1002/capr.12874>

Graham, J., Rodgers, J., & Cassidy, S. (2023). Why Are Autistic People More Likely to Experience Suicidal Thoughts? Applying the Integrated Motivational–Volitional Model with Autistic Adults. *Autism in Adulthood*. <https://doi.org/10.1089/aut.2023.0039>

Grant, A., Griffiths, C., Williams, K., & Brown, A. (2025). “I Just Gritted My Teeth to Get Through It All”: An Online Survey of Autistic People’s Experiences of Maternity Care in the United Kingdom. *Autism in Adulthood*. <https://doi.org/10.1089/aut.2024.0275>

Grant, A., Turner, S., Shaw, S. C. K., Williams, K., Morgan, H., Ellis, R., & Brown, A. (2024). “I am afraid of being treated badly if I show it”: A cross-sectional study of healthcare accessibility and Autism Health Passports among UK Autistic adults. *PLoS ONE*, *19*(5), e0303873. <https://doi.org/10.1371/journal.pone.0303873>

Grant, S., Norton, S., & Hoekstra, R. A. (2025). A Cross-Neurotype Analysis of Camouflaging and Illness Perceptions in People with Central Sensitivity Syndromes. *Autism in Adulthood*. <https://doi.org/10.1089/aut.2024.0186>

Green, R. M., Travers, A. M., Howe, Y., & McDougle, C. J. (2019). Women and Autism Spectrum Disorder: Diagnosis and Implications for Treatment of Adolescents and Adults. *Current Psychiatry Reports*, *21*(4). <https://doi.org/10.1007/s11920-019-1006-3>

Gurba, A. N., McNair, M. L., Hargreaves, A., Scheerer, N. E., Ng, C. S. M., & Lerner, M. D. (2024). Editorial: Break the stigma: autism. The future of research on autism stigma - towards multilevel, contextual & global understanding. *Frontiers in Psychiatry*, *15*. <https://doi.org/10.3389/fpsyt.2024.1504429>

Hake, R. (2025). Autistic trans camouflaging: an early phenomenological exploration. *Philosophical Psychology*, *38*(1), 150-167. <https://doi.org/10.1080/09515089.2024.2385056>

Halsall, J., Clarke, C., & Crane, L. (2021). “Camouflaging” by adolescent autistic girls who attend both mainstream and specialist resource classes: Perspectives of girls, their mothers and their educators. *Autism*, *25*(7), 2074-2086. <https://doi.org/10.1177/13623613211012819>

Han, E., Scior, K., Avramides, K., & Crane, L. (2022). A systematic review on autistic people's experiences of stigma and coping strategies. *Autism Res*, *15*(1), 12-26. <https://doi.org/10.1002/aur.2652>

Hannon, B., Mandy, W., & Hull, L. (2022). A comparison of methods for measuring camouflaging in autism. *Autism Research*, *16*(1). <https://doi.org/10.1002/aur.2850>

Harmens, M., Sedgewick, F., & Hobson, H. (2022). The Quest for Acceptance: A Blog-Based Study of Autistic Women's Experiences and Well-Being During Autism Identification and Diagnosis. *Autism Adulthood*, *4*(1), 42-51. <https://doi.org/10.1089/aut.2021.0016>

Harrop, C., Jones, D., Zheng, S., Nowell, S., Schultz, R., & Parish-Morris, J. (2019). Visual attention to faces in children with autism spectrum disorder: are there sex differences? *Molecular Autism*, *10*(1). <https://doi.org/10.1186/s13229-019-0276-2>

Harrop, C., Jones, D., Zheng, S., Nowell, S. W., Boyd, B. A., & Sasson, N. (2018). Sex differences in social attention in autism spectrum disorder. *Autism Research*, *11*(9), 1264-1275. <https://doi.org/10.1002/aur.1997>

Head, A. M., McGillivray, J. A., & Stokes, M. A. (2014). Gender differences in emotionality and sociability in children with autism spectrum disorders. *Molecular Autism*, *5*(1), 19. <https://doi.org/10.1186/2040-2392-5-19>

Hechler, F. C., Tuomainen, O., Weber, N., Fahr, F., Karlek, B., Maroske, M., Misia, M., & Caruana, N. (2025). "What does 'often' even mean?" Revising and validating the Comprehensive Autistic Trait Inventory in partnership with autistic people. *Molecular Autism*, *16*(1), 7. <https://doi.org/10.1186/s13229-025-00643-7>

Hennekam, S., Kulkarni, M., & Beatty, J. E. (2024). Neurodivergence and the Persistence of Neurotypical Norms and Inequalities in Educational and Occupational Settings. *Work, Employment and Society*, 09500170241255050. <https://doi.org/10.1177/09500170241255050>

Hernández, O., Durán, L. R., Fresno, A., & Chamorro, M. F. (2024). Camouflage in autism: a bibliometric perspective. *Advances in Autism*, *10*(4), 283-298. <https://doi.org/10.1108/AIA-01-2024-0007>

Hickey, A., Crabtree, J., & Stott, J. (2017). ‘Suddenly the first fifty years of my life made sense’: Experiences of older people with autism. *Autism*, *22*(3), 357-367. <https://doi.org/10.1177/1362361316680914>

Higgins, J. M., Arnold, S. R. C., Weise, J., Pellicano, E., & Trollor, J. N. (2021). Defining autistic burnout through experts by lived experience: Grounded Delphi method investigating #AutisticBurnout. *Autism*, *25*(8), 2356-2369. <https://doi.org/10.1177/13623613211019858>

Hill, J. (2024). ‘If I behave like the stupidly kind character maybe I will stop being accidentally rude to people’: does reading fiction inform the social understanding and masking behaviours of autistic females? *English in Education*, *0*(0), 1-16. <https://doi.org/10.1080/04250494.2024.2330905>

Holingue, C., Holmes, L. G., Cusano, J., & Rothman, E. F. (2025). Correlates of age at first alcohol use among a US-based sample of autistic underage youth. *Research in Autism Spectrum Disorders*, *119*, 102520. <https://doi.org/10.1016/j.rasd.2024.102520>

Hong, Y. H., Song, D.-Y., & Yoo, H. (2025). Sex differences in the prevalence of common comorbidities in autism: a narrative review. *Ewha Medical Journal*, *48*(1), e79-70. <https://doi.org/10.12771/emj.2024.e79>

Hongo, M., Oshima, F., Guan, S., Takahashi, T., Nitta, Y., Seto, M., Hull, L., Mandy, W., Ohtani, T., Tamura, M., & Shimizu, E. (2024). Reliability and validity of the Japanese version of the camouflaging autistic traits questionnaire. *Autism Research*, *17*(6), 1205-1217. <https://doi.org/10.1002/aur.3137>

Howe, S. J., Hull, L., Sedgewick, F., Hannon, B., & McMorris, C. A. (2023). Understanding camouflaging and identity in autistic children and adolescents using photo-elicitation. *Research in Autism Spectrum Disorders*, *108*, 102232. <https://doi.org/10.1016/j.rasd.2023.102232>

Huang, Y., Arnold, S. R., Foley, K.-R., & Trollor, J. N. (2020). Diagnosis of autism in adulthood: A scoping review. *Autism*, 136236132090312. <https://doi.org/10.1177/1362361320903128>

Huang, Y., Hwang, Y. I., Arnold, S. R. C., Lawson, L. P., Richdale, A. L., & Trollor, J. N. (2022). Autistic Adults’ Experiences of Diagnosis Disclosure. *Journal of Autism and Developmental Disorders*, *52*(12), 5301-5307. <https://doi.org/10.1007/s10803-021-05384-z>

Huang, Y., Trollor, J. N., Foley, K.-R., & Arnold, S. R. C. (2023). “I've Spent My Whole Life Striving to Be Normal”: Internalized Stigma and Perceived Impact of Diagnosis in Autistic Adults. *Autism in Adulthood*. <https://doi.org/10.1089/aut.2022.0066>

Hughes, R., Curley, K., & Kotera, Y. (2024). Parents’ Experiences after Their Child’s Autism Diagnosis: A Reflexive Thematic Analysis. *Psychiatry International*, *5*(3), 370-394.

Hull, L., Lai, M.-C., Baron-Cohen, S., Allison, C., Smith, P., Petrides, K., & Mandy, W. (2020). Gender differences in self-reported camouflaging in autistic and non-autistic adults. *Autism*, *24*(2), 352-363. <https://doi.org/10.1177/1362361319864804>

Hull, L., Levy, L., Lai, M.-C., Petrides, K. V., Baron-Cohen, S., Allison, C., Smith, P., & Mandy, W. (2021). Is social camouflaging associated with anxiety and depression in autistic adults? *Molecular Autism*, *12*(1). <https://doi.org/10.1186/s13229-021-00421-1>

Hull, L., & Mandy, W. (2017). Protective effect or missed diagnosis? Females with autism spectrum disorder. *Future Neurology*, *12*(3), 159-169. <https://doi.org/10.2217/fnl-2017-0006>

Hull, L., Mandy, W., Belcher, H., & Petrides, K. V. (2024). Validation of the camouflaging autistic traits questionnaire short form (CATQ-SF). *Comprehensive Psychiatry*, *135*, 152525. <https://doi.org/10.1016/j.comppsych.2024.152525>

Hull, L., Mandy, W., Lai, M.-C., Baron-Cohen, S., Allison, C., Smith, P., & Petrides, K. V. (2019). Development and Validation of the Camouflaging Autistic Traits Questionnaire (CAT-Q). *Journal of Autism and Developmental Disorders*, *49*(3), 819-833. <https://doi.org/10.1007/s10803-018-3792-6>

Hull, L., Petrides, K. V., Allison, C., Smith, P., Baron-Cohen, S., Lai, M.-C., & Mandy, W. (2017). “Putting on My Best Normal”: Social Camouflaging in Adults with Autism Spectrum Conditions. *Journal of Autism and Developmental Disorders*, *47*(8), 2519-2534. <https://doi.org/10.1007/s10803-017-3166-5>

Hull, L., Petrides, K. V., & Mandy, W. (2020). Cognitive Predictors of Self‐Reported Camouflaging in Autistic Adolescents. *Autism Research*. <https://doi.org/10.1002/aur.2407>

Humphrey, N., & Lewis, S. (2008). `Make me normal':The views and experiences of pupils on the autistic spectrum in mainstream secondary schools. *Autism*, *12*(1), 23-46. <https://doi.org/10.1177/1362361307085267>

Husmann, J., Feller, C., Ilen, L., & Schneider, M. (2024). A Multi-Method Approach for the Identification of Social Functioning Profiles in Autistic Adolescents and Young Adults Without Intellectual Disability. *Journal of Autism and Developmental Disorders*. <https://doi.org/10.1007/s10803-024-06607-9>

Iacomini, S., Fabiani, M., Serafini, E., & Tirelli, V. (2024). Exploring experiences and potential of neurodivergent women entrepreneurs: a qualitative study. *Journal of Clinical & Developmental Psychology*, *6*(2-3). <https://doi.org/10.13129/2612-4033/0110-4276>

Jarman, B., & Rayner, C. (2015). Asperger's and Girls: What Teachers Need to Know. *Australasian Journal of Special Education*, *39*(2), 128-142. <https://doi.org/10.1017/jse.2015.7>

Jedrzejewska, A., & Dewey, J. (2022). Camouflaging in Autistic and Non-autistic Adolescents in the Modern Context of Social Media. *Journal of Autism and Developmental Disorders*, *52*(2), 630-646. <https://doi.org/10.1007/s10803-021-04953-6>

Jellett, R., & Muggleton, J. (2022). Implications of Applying “Clinically Significant Impairment” to Autism Assessment: Commentary on Six Problems Encountered in Clinical Practice. *Journal of Autism and Developmental Disorders*, *52*(3), 1412-1421. <https://doi.org/10.1007/s10803-021-04988-9>

Jensen, M. K., Lydiksen, T. W., Pedersen, S. H., & Bentz, M. (2024). Compensatory Strategies in Adolescent Females with a Restrictive Eating Disorder and Later Diagnosed Autism: A Qualitative Study of Patient Records. *Journal of Psychiatry and Brain Science*, *9*(6), e240009, Article e240009. <https://doi.org/10.20900/jpbs.20240009>

Johnson, T. D., & Joshi, A. (2014). Disclosure on the Spectrum: Understanding Disclosure Among Employees on the Autism Spectrum. *Industrial and Organizational Psychology*, *7*(2), 278-281. <https://doi.org/10.1111/iops.12149>

Johnson, T. D., & Joshi, A. (2016). Dark clouds or silver linings? A stigma threat perspective on the implications of an autism diagnosis for workplace well-being. *J Appl Psychol*, *101*(3), 430-449. <https://doi.org/10.1037/apl0000058>

Jolliffe, R., Adams, D., & Simpson, K. (2025). State and trait anxiety in autistic children; Signs reported by autistic adults and parents of autistic children. *Research in Autism*, *123*, 202552. <https://doi.org/10.1016/j.reia.2025.202552>

Jorgenson, C., Lewis, T., Rose, C., & Kanne, S. (2020). Social Camouflaging in Autistic and Neurotypical Adolescents: A Pilot Study of Differences by Sex and Diagnosis. *Journal of Autism and Developmental Disorders*. <https://doi.org/10.1007/s10803-020-04491-7>

Kaat, A. J., Shui, A. M., Ghods, S. S., Farmer, C. A., Esler, A. N., Thurm, A., Georgiades, S., Kanne, S. M., Lord, C., Kim, Y. S., & Bishop, S. L. (2020). Sex differences in scores on standardized measures of autism symptoms: a multisite integrative data analysis. *Journal of Child Psychology and Psychiatry*. <https://doi.org/10.1111/jcpp.13242>

Kanfiszer, L., Davies, F., & Collins, S. (2017). ‘I was just so different’: The experiences of women diagnosed with an autism spectrum disorder in adulthood in relation to gender and social relationships. *Autism*, *21*(6), 661-669. <https://doi.org/10.1177/1362361316687987>

Kapp, S. K., Steward, R., Crane, L., Elliott, D., Elphick, C., Pellicano, E., & Russell, G. (2019). ‘People should be allowed to do what they like’: Autistic adults’ views and experiences of stimming. *Autism*, *23*(7), 1782-1792. <https://doi.org/10.1177/1362361319829628>

Karaminis, T., Gabrielatos, C., Maden-Weinberger, U., & Beattie, G. (2024). Gender and family-role portrayals of autism in British newspapers: An intersectional corpus-based study. *Autism*, 13623613241303547. <https://doi.org/10.1177/13623613241303547>

Keating, C. T., Hickman, L., Geelhand, P., Takahashi, T., Leung, J., Monk, R., Schuster, B., Rybicki, A., Girolamo, T. M., Clin, E., Papastamou, F., Belenger, M., Eigsti, I.-M., Cook, J. L., Kosaka, H., Osu, R., Okamoto, Y., & Sowden-Carvalho, S. (2024). Cross-cultural variation in experiences of acceptance, camouflaging and mental health difficulties in autism: A registered report. *PLoS ONE*, *19*(3), e0299824. <https://doi.org/10.1371/journal.pone.0299824>

Kentrou, V., Oostervink, M., Scheeren, A. M., & Begeer, S. (2021). Stability of co-occurring psychiatric diagnoses in autistic men and women. *Research in Autism Spectrum Disorders*, *82*, 101736. <https://doi.org/10.1016/j.rasd.2021.101736>

Khudiakova, V., Alexandrovsky, M., Ai, W., & Lai, M.-C. (2024). What We Know and Do Not Know About Camouflaging, Impression Management, and Mental Health and Wellbeing in Autistic People. *Autism Research*, *18*(2). <https://doi.org/10.1002/aur.3299>

Khudiakova, V., Le Forestier, J. M., & Chasteen, A. L. (2024). To mask or not to mask: The role of concealment behavior, stigma experience, and community connectedness in autistic people's mental health. *Neurodiversity*, *2*, 27546330241255121. <https://doi.org/10.1177/27546330241255121>

Khudiakova, V., Levy, X., Sowden-Carvalho, S., & Surtees, A. D. R. (2025). A qualitative exploration of autistic people's experiences of camouflaging across different social contexts. *Autism in Adulthood*.

Khudiakova, V., Russell, E., Sowden-Carvalho, S., & Surtees, A. D. R. (2024). A systematic review and meta-analysis of mental health outcomes associated with camouflaging in autistic people. *Research in Autism Spectrum Disorders*, *118*, 102492. <https://doi.org/10.1016/j.rasd.2024.102492>

Kirkovski, M., Enticott, P. G., & Fitzgerald, P. B. (2013). A Review of the Role of Female Gender in Autism Spectrum Disorders. *Journal of Autism and Developmental Disorders*, *43*(11), 2584-2603. <https://doi.org/10.1007/s10803-013-1811-1>

Klein, J., Krahn, R., Howe, S., Lewis, J., McMorris, C., & Macoun, S. (2024). A systematic review of social camouflaging in autistic adults and youth: Implications and theory. *Development and Psychopathology*, 1-15. <https://doi.org/10.1017/S0954579424001159>

Klein, J., & Macoun, S. J. (2025). Person-environment fit and social camouflaging in autism. *New Ideas in Psychology*, *76*, 101112. <https://doi.org/10.1016/j.newideapsych.2024.101112>

Knutsen, J., Crossman, M., Perrin, J., Shui, A., & Kuhlthau, K. (2019). Sex differences in restricted repetitive behaviors and interests in children with autism spectrum disorder: An Autism Treatment Network study. *Autism*, *23*(4), 858-868. <https://doi.org/10.1177/1362361318786490>

Kong, H., Xie, J., Dou, F., Li, X., XinyuWang, & Huang, Y. (2024). Autistic traits and depressive symptoms: The mediation chain path of social camouflaging to self-concept clarity and social camouflaging to self-disgust. In: PsyArXiv.

Koteyko, N., Van Driel, M., Billan, S., Barros Pena, B., & Vines, J. (2024). Stigma Management Strategies of Autistic Social Media Users. *Autism in Adulthood*. <https://doi.org/10.1089/aut.2023.0095>

Koteyko, N., van Driel, M., & Vines, J. (2022). Autistic sociality on Twitter: Enacted affordances and affiliation strategies. *Discourse & Communication*, *16*(4), 385-402. <https://doi.org/10.1177/17504813211070655>

Krishnan, S. G., Cohn, E. S., & Orsmond, G. I. (2024). The Transactional Nature of Autistic Young Adults’ Sensory and Social Experiences: Negotiating Young Adulthood. *Autism in Adulthood*. <https://doi.org/10.1089/aut.2024.0185>

Kuo, Y.-C., Ni, H.-C., & Liu, C.-H. (2024). The associations between self-rated autistic traits, social camouflaging, and mental health outcomes in Taiwanese anime, comics and games (ACG) doujin creators: an exploratory study. *BMC Psychology*, *12*(1), 531. <https://doi.org/10.1186/s40359-024-02019-7>

Kuzminski, R., Bölte, S., Lawson, W., Falkmer, M., Black, M. H., Girdler, S., & Milbourn, B. (2024). A Coproduced Exploration of Factors Influential to Quality of Life from the Perspective of Autistic Adults. *Autism in Adulthood*. <https://doi.org/10.1089/aut.2023.0091>

Lai, M.-C., Amestoy, A., Bishop, S., Brown, H. M., Giwa Onaiwu, M., Halladay, A., Harrop, C., Hotez, E., Huerta, M., Kelly, A., Miller, D., Nordahl, C. W., Ratto, A. B., Saulnier, C., Siper, P. M., Sohl, K., Zwaigenbaum, L., & Goldman, S. (2023). Improving autism identification and support for individuals assigned female at birth: clinical suggestions and research priorities. *The Lancet Child & Adolescent Health*, *7*(12), 897-908. <https://doi.org/10.1016/S2352-4642(23)00221-3>

Lai, M.-C., & Baron-Cohen, S. (2015). Identifying the lost generation of adults with autism spectrum conditions. *The Lancet Psychiatry*, *2*(11), 1013-1027. <https://doi.org/10.1016/s2215-0366(15)00277-1>

Lai, M.-C., Lin, H.-Y., & Ameis, S. H. (2022). Towards equitable diagnoses for autism and attention-deficit/hyperactivity disorder across sexes and genders. *Current Opinion in Psychiatry*, *35*(2), 90-100. <https://doi.org/10.1097/YCO.0000000000000770>

Lai, M.-C., Lombardo, M. V., Auyeung, B., Chakrabarti, B., & Baron-Cohen, S. (2015). Sex/Gender Differences and Autism: Setting the Scene for Future Research. *Journal of the American Academy of Child & Adolescent Psychiatry*, *54*(1), 11-24. <https://doi.org/10.1016/j.jaac.2014.10.003>

Lai, M.-C., Lombardo, M. V., Chakrabarti, B., Ruigrok, A. N., Bullmore, E. T., Suckling, J., Auyeung, B., Happé, F., Szatmari, P., Baron-Cohen, S., Bailey, A. J., Bolton, P. F., Carrington, S., Catani, M., Craig, M. C., Daly, E. M., Deoni, S. C., Ecker, C., Henty, J., . . . Williams, S. C. (2019). Neural self-representation in autistic women and association with ‘compensatory camouflaging’. *Autism*, *23*(5), 1210-1223. <https://doi.org/10.1177/1362361318807159>

Lai, M.-C., Lombardo, M. V., Pasco, G., Ruigrok, A. N. V., Wheelwright, S. J., Sadek, S. A., Chakrabarti, B., & Baron-Cohen, S. (2011). A Behavioral Comparison of Male and Female Adults with High Functioning Autism Spectrum Conditions. *PLoS ONE*, *6*(6), e20835. <https://doi.org/10.1371/journal.pone.0020835>

Lai, M.-C., Lombardo, M. V., Ruigrok, A. N., Chakrabarti, B., Auyeung, B., Szatmari, P., Happé, F., & Baron-Cohen, S. (2017). Quantifying and exploring camouflaging in men and women with autism. *Autism*, *21*(6), 690-702. <https://doi.org/10.1177/1362361316671012>

Lai, M.-C., & Szatmari, P. (2020). Sex and gender impacts on the behavioural presentation and recognition of autism. *Current Opinion in Psychiatry*, *33*(2), 117-123. <https://doi.org/10.1097/yco.0000000000000575>

Lam, G. Y. H., Chow, C. K. C., & Chan, S. W. (2024). A qualitative exploration of the experience of autistic females in Hong Kong. *Autism*, 13623613241295318. <https://doi.org/10.1177/13623613241295318>

Lawson, L. P. (2019). Sex Differences in Autism Spectrum Disorders Across the Lifespan. *Current Developmental Disorders Reports*, *6*(2), 57-66. <https://doi.org/10.1007/s40474-019-00164-y>

Lawson, L. P., Joshi, R., Barbaro, J., & Dissanayake, C. (2018). Gender Differences During Toddlerhood in Autism Spectrum Disorder: A Prospective Community-Based Longitudinal Follow-Up Study. *Journal of Autism and Developmental Disorders*, *48*(8), 2619-2628. <https://doi.org/10.1007/s10803-018-3516-y>

Lawson, W. B. (2020). Adaptive Morphing and Coping with Social Threat in Autism: An Autistic Perspective. *Journal of Intellectual Disability - Diagnosis and Treatment*, *8*(3), 519-526. <https://doi.org/10.6000/2292-2598.2020.08.03.29>

Leadbitter, K., Buckle, K. L., Ellis, C., & Dekker, M. (2021). Autistic Self-Advocacy and the Neurodiversity Movement: Implications for Autism Early Intervention Research and Practice. *Frontiers in Psychology*, *12*. <https://doi.org/10.3389/fpsyg.2021.635690>

Leaf, J. B., Creem, A. N., Bukszpan, A., Hickey, J., & Hillhouse, B. (2023). On the Status and Knowledge of Camouflaging, Masking, and Compensatory Behaviors in Autism Spectrum Disorder. *Education and Training in Autism and Developmental Disabilities*, *58*(3), 283-298.

Leedham, A., Thompson, A. R., Smith, R., & Freeth, M. (2020). ‘I was exhausted trying to figure it out’: The experiences of females receiving an autism diagnosis in middle to late adulthood. *Autism*, *24*(1), 135-146. <https://doi.org/10.1177/1362361319853442>

Lehnhardt, F.-G., Falter, C. M., Gawronski, A., Pfeiffer, K., Tepest, R., Franklin, J., & Vogeley, K. (2016). Sex-Related Cognitive Profile in Autism Spectrum Disorders Diagnosed Late in Life: Implications for the Female Autistic Phenotype. *Journal of Autism and Developmental Disorders*, *46*(1), 139-154. <https://doi.org/10.1007/s10803-015-2558-7>

Lei, J., Cooper, K., & Hollocks, M. J. (2024). Psychological Interventions for Autistic Adolescents with Co-Occurring Anxiety and Depression: Considerations Linked to Autism Social Identity and Masking. *Autism in Adulthood*. <https://doi.org/10.1089/aut.2024.0005>

Lei, J., Leigh, E., Charman, T., Russell, A., & Hollocks, M. J. (2024a). Exploring the association between social camouflaging and self- versus caregiver-report discrepancies in anxiety and depressive symptoms in autistic and non-autistic socially anxious adolescents. *Autism*, *28*(10), 2657-2674. <https://doi.org/10.1177/13623613241238251>

Lei, J., Leigh, E., Charman, T., Russell, A., & Hollocks, M. J. (2024b). Understanding the relationship between social camouflaging in autism and safety behaviours in social anxiety in autistic and non-autistic adolescents. *Journal of Child Psychology and Psychiatry*, *65*(3), 285-297. <https://doi.org/10.1111/jcpp.13884>

Lei, J., Mason, C., Russell, A., Hollocks, M. J., & Leigh, E. (2024). Understanding Mechanisms that Maintain Social Anxiety Disorder in Autistic Individuals Through the Clark and Wells (1995) Model and Beyond: A Systematic Review. *Clinical Child and Family Psychology Review*, *27*(4), 966-1030. <https://doi.org/10.1007/s10567-024-00509-z>

Lei, J., & Nocon, A. S. (2024). Are we Missing Character in Strengths-Based Approaches to Coaching and Therapy for Autistic People? *Autism in Adulthood*. <https://doi.org/10.1089/aut.2024.0227>

Lei, J., Qian, X., & Kim, K. M. (2025). Social Network Structure in Autistic Individuals: A Systematic Review. *Autism in Adulthood*. <https://doi.org/10.1089/aut.2024.0029>

Lewis, L. F. (2017). A Mixed Methods Study of Barriers to Formal Diagnosis of Autism Spectrum Disorder in Adults. *Journal of Autism and Developmental Disorders*, *47*(8), 2410-2424. <https://doi.org/10.1007/s10803-017-3168-3>

Lewis, L. F., & Stevens, K. (2023). The lived experience of meltdowns for autistic adults. *Autism*, 13623613221145783. <https://doi.org/10.1177/13623613221145783>

Libsack, E. J., Keenan, E. G., Freden, C. E., Mirmina, J., Iskhakov, N., Krishnathasan, D., & Lerner, M. D. (2021). A Systematic Review of Passing as Non-autistic in Autism Spectrum Disorder. *Clinical Child and Family Psychology Review*, *24*(4), 783-812. <https://doi.org/10.1007/s10567-021-00365-1>

Lilley, R., Lawson, W., Hall, G., Mahony, J., Clapham, H., Heyworth, M., Arnold, S., Trollor, J., Yudell, M., & Pellicano, E. (2022). “Peas in a pod”: Oral History Reflections on Autistic Identity in Family and Community by Late-Diagnosed Adults. *Journal of Autism and Developmental Disorders*. <https://doi.org/10.1007/s10803-022-05667-z>

Lilley, R., Lawson, W., Hall, G., Mahony, J., Clapham, H., Heyworth, M., Arnold, S. R. C., Trollor, J. N., Yudell, M., & Pellicano, E. (2021). ‘A way to be me’: Autobiographical reflections of autistic adults diagnosed in mid-to-late adulthood. *Autism*, *26*(6), 1395-1408. <https://doi.org/10.1177/13623613211050694>

Lindsay, S., Osten, V., Rezai, M., & Bui, S. (2019). Disclosure and workplace accommodations for people with autism: a systematic review. *Disability and Rehabilitation*, 1-14. <https://doi.org/10.1080/09638288.2019.1635658>

Livingston, L. A., Colvert, E., Bolton, P., & Happé, F. (2019). Good social skills despite poor theory of mind: exploring compensation in autism spectrum disorder. *Journal of Child Psychology and Psychiatry*, *60*(1), 102-110. <https://doi.org/10.1111/jcpp.12886>

Livingston, L. A., & Happé, F. (2017). Conceptualising compensation in neurodevelopmental disorders: Reflections from autism spectrum disorder. *Neuroscience & Biobehavioral Reviews*, *80*, 729-742. <https://doi.org/10.1016/j.neubiorev.2017.06.005>

Livingston, L. A., Shah, P., & Happé, F. (2019). Compensatory strategies below the behavioural surface in autism: a qualitative study. *The Lancet Psychiatry*, *6*(9), 766-777. <https://doi.org/10.1016/s2215-0366(19)30224-x>

Livingston, L. A., Shah, P., Milner, V., & Happé, F. (2020). Quantifying compensatory strategies in adults with and without diagnosed autism. *Molecular Autism*, *11*(1). <https://doi.org/10.1186/s13229-019-0308-y>

Long, H., Cooper, K., & Russell, A. (2024). ‘Autism is the Arena and OCD is the Lion’: Autistic adults’ experiences of co-occurring obsessive-compulsive disorder and repetitive restricted behaviours and interests. *Autism*, *28*(11), 2897-2908. <https://doi.org/10.1177/13623613241251512>

Loo, B. R. Y., Teo, T. J. Y., Liang, M. J., Leong, D.-J., Tan, D. W., Zhuang, S., Hull, L., Livingston, L. A., Mandy, W., Happé, F., & Magiati, I. (2023). Exploring autistic adults’ psychosocial experiences affecting beginnings, continuity and change in camouflaging over time: A qualitative study in Singapore. *Autism*, 13623613231180075. <https://doi.org/10.1177/13623613231180075>

Loomes, R., Hull, L., & Mandy, W. P. L. (2017). What Is the Male-to-Female Ratio in Autism Spectrum Disorder? A Systematic Review and Meta-Analysis. *Journal of the American Academy of Child & Adolescent Psychiatry*, *56*(6), 466-474. <https://doi.org/10.1016/j.jaac.2017.03.013>

Lorenz, S., & Hull, L. (2024). Do All of Us Camouflage? Exploring Levels of Camouflaging and Mental Health Well-Being in the General Population. *Trends in Psychology*. <https://doi.org/10.1007/s43076-024-00357-4>

Lu, M., Pang, F., Peng, T., Liu, Y., & Wang, R. (2023). ‘Struggling to appear normal’: a moderated mediational analysis of empathy and camouflaging in the association between autistic traits and depressive symptoms. *International Journal of Developmental Disabilities*, 1-11. <https://doi.org/10.1080/20473869.2023.2181273>

Lundin, K., Mahdi, S., Isaksson, J., & Bölte, S. (2020). Functional gender differences in autism: An international, multidisciplinary expert survey using the International Classification of Functioning, Disability, and Health model. *Autism*, *25*(4), 1020-1035. <https://doi.org/10.1177/1362361320975311>

Lynch, J., Murphy, A., & Cezar Da Cruz, D. (2024). A phenomenological exploration of the occupational identity of late-diagnosed autistic women in the United Kingdom. *Discover Psychology*, *4*(1), 123. <https://doi.org/10.1007/s44202-024-00234-2>

Mademtzi, M., Singh, P., Shic, F., & Koenig, K. (2018). Challenges of Females with Autism: A Parental Perspective. *Journal of Autism and Developmental Disorders*, *48*(4), 1301-1310. <https://doi.org/10.1007/s10803-017-3341-8>

Mahony, C., & O'Ryan, C. (2022). A molecular framework for autistic experiences: Mitochondrial allostatic load as a mediator between autism and psychopathology. *Frontiers in Psychiatry*, *13*. <https://doi.org/10.3389/fpsyt.2022.985713>

Mandy, W., & Lai, M.-C. (2017). Towards sex- and gender-informed autism research. *Autism*, *21*(6), 643-645. <https://doi.org/10.1177/1362361317706904>

Mandy, W., & Tchanturia, K. (2015). Do women with eating disorders who have social and flexibility difficulties really have autism? A case series. *Molecular Autism*, *6*(1), 6. <https://doi.org/10.1186/2040-2392-6-6>

Mantzalas, J., Richdale, A. L., Adikari, A., Lowe, J., & Dissanayake, C. (2022). What Is Autistic Burnout? A Thematic Analysis of Posts on Two Online Platforms. *Autism Adulthood*, *4*(1), 52-65. <https://doi.org/10.1089/aut.2021.0021>

Mantzalas, J., Richdale, A. L., & Dissanayake, C. (2022). A conceptual model of risk and protective factors for autistic burnout. *Autism Research*, *15*(6), 976-987. <https://doi.org/10.1002/aur.2722>

Mantzalas, J., Richdale, A. L., Li, X., & Dissanayake, C. (2024). Measuring and validating autistic burnout. *Autism Research*, *17*(7), 1417-1449. <https://doi.org/10.1002/aur.3129>

Marocchini, E. (2023). Impairment or difference? The case of Theory of Mind abilities and pragmatic competence in the Autism Spectrum. *Applied Psycholinguistics*, *44*(3), 365-383. <https://doi.org/10.1017/S0142716423000024>

Mazurek, M. O., Pappagianopoulos, J., Brunt, S., Nevill, R., Menezes, M., Burroughs, C., Sadikova, E., Smith, J. V., & Howard, M. (2024). Inner experiences, mental health, and well-being in autistic and non-autistic adults. *Autism Research*, *17*(12), 2676-2688. <https://doi.org/10.1002/aur.3273>

McAuliffe, C., Walsh, R. J., & Cage, E. (2022). “My whole life has been a process of finding labels that fit”: A Thematic Analysis of Autistic LGBTQIA+ Identity and Inclusion in the LGBTQIA+ Community. *Autism in Adulthood*. <https://doi.org/10.1089/aut.2021.0074>

McFayden, T. C., Putnam, O., Grzadzinski, R., & Harrop, C. (2023). Sex Differences in the Developmental Trajectories of Autism Spectrum Disorder. *Current Developmental Disorders Reports*, *10*(1), 80-91. <https://doi.org/10.1007/s40474-023-00270-y>

McKinney, A., O’Brien, S., Maybin, J. A., Chan, S. W. Y., Richer, S., & Rhodes, S. (2024). Camouflaging in neurodivergent and neurotypical girls at the transition to adolescence and its relationship to mental health: A participatory methods research study. *JCPP Advances*, *4*(4), e12294. <https://doi.org/10.1002/jcv2.12294>

McKinnon, K., Bougoure, M., Zhuang, S., Tan, D. W., & Magiati, I. (2024). Exploring the construct validity of the Camouflaging Autistic Traits Questionnaire: A factor analytic study. *Autism*, 13623613241287964. <https://doi.org/10.1177/13623613241287964>

McMahon, C. M., Henry, S., & Linthicum, M. (2020). Employability in autism spectrum disorder (ASD): Job candidate’s diagnostic disclosure and asd characteristics and employer’s ASD knowledge and social desirability. *Journal of Experimental Psychology: Applied*. <https://doi.org/10.1037/xap0000282>

McMorris, C. A., Turner, K., Johnston, K., Clark, C. A., Howe, S. J., McConnell, M., & McFee, K. (2024). Facing Your Fears in autistic youth with co-occurring psychiatric conditions: Reductions in symptoms of anxiety and depression. *Research in Autism Spectrum Disorders*, *117*, 102463. <https://doi.org/10.1016/j.rasd.2024.102463>

McMurtry, C., Freeman, C., Perkins, J., Donnelly, G. M., & Moore, I. S. (2025). Developing inclusive policy and guidelines in sport: a call to action for sport governing bodies and individuals to support neurodivergent athletes. *British Journal of Sports Medicine*, *59*(6), 355. <https://doi.org/10.1136/bjsports-2024-108989>

McQuaid, G. A., Sadowski, L. Y., Lee, N. R., & Wallace, G. L. (2023). An Examination of Perceived Stress and Emotion Regulation Challenges as Mediators of Associations Between Camouflaging and Internalizing Symptomatology. *Autism in Adulthood*. <https://doi.org/10.1089/aut.2022.0121>

Mesa, S., & Hamilton, L. G. (2022). “We are different, that’s a fact, but they treat us like we’re different-er”: understandings of autism and adolescent identity development. *Advances in Autism*, *8*(3), 217-231. <https://doi.org/10.1108/AIA-12-2020-0071>

Miller, D., Rees, J., & Pearson, A. (2021). “Masking Is Life”: Experiences of Masking in Autistic and Nonautistic Adults. *Autism in Adulthood*, *3*(4), 330-338. <https://doi.org/10.1089/aut.2020.0083>

Milner, V., Colvert, E., Hull, L., Cook, J., Ali, D., Mandy, W., & Happé, F. (2023). Does camouflaging predict age at autism diagnosis? A comparison of autistic men and women. *Autism Research*, *n/a*(n/a). <https://doi.org/10.1002/aur.3059>

Milner, V., Colvert, E., Mandy, W., & Happé, F. (2022). A comparison of self-report and discrepancy measures of camouflaging: Exploring sex differences in diagnosed autistic versus high autistic trait young adults. *Autism Research*, *16*(3). <https://doi.org/10.1002/aur.2873>

Milner, V., Mandy, W., Happé, F., & Colvert, E. (2022). Sex differences in predictors and outcomes of camouflaging: Comparing diagnosed autistic, high autistic trait and low autistic trait young adults. *Autism*, *0*(0), 13623613221098240. <https://doi.org/10.1177/13623613221098240>

Milner, V., McIntosh, H., Colvert, E., & Happé, F. (2019). A Qualitative Exploration of the Female Experience of Autism Spectrum Disorder (ASD). *Journal of Autism and Developmental Disorders*, *49*(6), 2389-2402. <https://doi.org/10.1007/s10803-019-03906-4>

Milton, D., & Lyte, M. (2012). The normalisation agenda and the psycho-emotional disablement of autistic people. *Autonomy, the Critical Journal of Interdisciplinary Autism Studies*, *1*(1).

Milton, D., & Sims, T. (2016). How is a sense of well-being and belonging constructed in the accounts of autistic adults? *Disability & Society*, *31*(4), 520-534. <https://doi.org/10.1080/09687599.2016.1186529>

Miranda-Ojeda, R., Wickramasinghe, A., Ntolkeras, G., Castanho, I., & Yassin, W. (2025). The Neurodiversity Framework in Medicine: On the Spectrum. *Developmental Neurobiology*, *85*(1), e22960. <https://doi.org/10.1002/dneu.22960>

Mogensen, L., & Mason, J. (2015). The meaning of a label for teenagers negotiating identity: experiences with autism spectrum disorder. *Sociology of Health & Illness*, *37*(2), 255-269. <https://doi.org/10.1111/1467-9566.12208>

Moore, H. L., Cassidy, S., & Rodgers, J. (2023). Exploring the mediating effect of camouflaging and the moderating effect of autistic identity on the relationship between autistic traits and mental wellbeing. *Autism Research*, *n/a*(n/a). <https://doi.org/10.1002/aur.3073>

Morris, I. F., Sykes, J. R., Paulus, E. R., Dameh, A., Razzaque, A., Esch, L. V., Gruenig, J., & Zelazo, P. D. (2025). Beyond self-regulation: Autistic experiences and perceptions of stimming. *Neurodiversity*, *3*, 27546330241311096. <https://doi.org/10.1177/27546330241311096>

Morrison, K. E., DeBrabander, K. M., Faso, D. J., & Sasson, N. J. (2019). Variability in first impressions of autistic adults made by neurotypical raters is driven more by characteristics of the rater than by characteristics of autistic adults. *Autism*, *23*(7), 1817-1829. <https://doi.org/10.1177/1362361318824104>

Moseley, R. L., Hitchiner, R., & Kirkby, J. A. (2018). Self-reported sex differences in high-functioning adults with autism: a meta-analysis. *Molecular Autism*, *9*(1). <https://doi.org/10.1186/s13229-018-0216-6>

Mosquera, M. L., Mandy, W., Pavlopoulou, G., & Dimitriou, D. (2021). Autistic adults’ personal experiences of navigating a social world prior to and during Covid-19 lockdown in Spain. *Research in Developmental Disabilities*, *117*, 104057. <https://doi.org/10.1016/j.ridd.2021.104057>

Mosquera, M. L., Zubizarreta, S. C.-P., Fungueiriño, M. T., Navarro, M. A., Hull, L., Álvarez, A. C., Gafo, B. G., & Prieto, M. F. (2022). Assessing Camouflaging in the Spanish Population: Cultural Adaptation of the Camouflaging Autistic Traits Questionnaire for Spain. <https://doi.org/10.21203/rs.3.rs-2048328/v1>

Muggleton, J. T. B., MacMahon, K., & Johnston, K. (2019). Exactly the same but completely different: A thematic analysis of Clinical Psychologists’ conceptions of Autism across genders. *Research in Autism Spectrum Disorders*, *62*, 75-84. <https://doi.org/10.1016/j.rasd.2019.03.004>

Muratori, F., Tancredi, R., & Calderoni, S. (2024). Insights from pioneers of autism. *Italian Journal of Psychiatry*, *10*(2), 68-71. <https://doi.org/10.36180/2421-4469-2024-622>

Murphy, S., Flower, R. L., & Jellett, R. (2022). Women seeking an autism diagnosis in Australia: A qualitative exploration of factors that help and hinder. *Autism*, 13623613221117911. <https://doi.org/10.1177/13623613221117911>

Mussey, J. L., Ginn, N. C., & Klinger, L. G. (2017). Are males and females with autism spectrum disorder more similar than we thought? *Autism*, *21*(6), 733-737. <https://doi.org/10.1177/1362361316682621>

Myles, O., Boyle, C., & Richards, A. (2019). The social experiences and sense of belonging in adolescent females with autism in mainstream school. *Educational & Child Psychology*, *36*(4), 8-21. <https://doi.org/10.53841/bpsecp.2019.36.4.8>

Nagar Shimoni, H., Zilbershot Fink, E., & Leitner, Y. (2025). The Clinical Phenotype of Early Selective Mutism and Later Autism Spectrum Disorder in Girls: A Case Series Analysis. *Children*, *12*(2).

Ng, C. S. M., & Ng, S. S. L. (2022). A qualitative study on the experience of stigma for Chinese parents of children with autism spectrum disorder. *Scientific Reports*, *12*(1), 19550. <https://doi.org/10.1038/s41598-022-23978-0>

Nieradka, F., & Kossewska, J. (2023). Camouflage, self-esteem and gratitude in young adults with varying degrees of autistic traits in Poland. *International Journal of Special Education (IJSE)*, *38*(2), 188-198. <https://doi.org/10.52291/ijse.2023.38.33>

Norvaišaite, A., & Tateo, L. (2024). Development of performative identities in autism writers’ autobiographies. *European Journal of Special Needs Education*, 1-15. <https://doi.org/10.1080/08856257.2024.2407660>

O'Connor, C., Kadianaki, I., Maunder, K., & McNicholas, F. (2018). How does psychiatric diagnosis affect young people's self-concept and social identity? A systematic review and synthesis of the qualitative literature. *Social Science & Medicine*, *212*, 94-119. <https://doi.org/10.1016/j.socscimed.2018.07.011>

O'Loghlen, J. J., & Lang, C. P. (2023). High Autistic Traits or Low Social Competence? Correlates of Social Camouflaging in Non-Autistic Adults. *Autism in Adulthood*. <https://doi.org/10.1089/aut.2022.0094>

O’Connor, C., Burke, J., & Rooney, B. (2020). Diagnostic Disclosure and Social Marginalisation of Adults with ASD: Is There a Relationship and What Mediates It? *Journal of Autism and Developmental Disorders*, *50*(9), 3367-3379. <https://doi.org/10.1007/s10803-019-04239-y>

Okamoto, C., Valle, D., Paes, A., Chemin, A., Ferreira, J., Luz, L. G., Nakanishi, M. A., & Santos, V. (2024). Camouflage mechanisms in Brazilian children with autistic spectrum disorder: is there a difference between genders? *International Journal of Developmental Disabilities*, 1-8. <https://doi.org/10.1080/20473869.2023.2294391>

Oliver, M., Poysden, Z., & Gillespie-Smith, K. (2024). A Qualitative Systematic Review and Meta-synthesis of Mothers’ Experiences of Parenting Autistic Women and Girls. *Review Journal of Autism and Developmental Disorders*. <https://doi.org/10.1007/s40489-024-00472-z>

Ormond, S., Brownlow, C., Garnett, M. S., Rynkiewicz, A., & Attwood, T. (2018). Profiling Autism Symptomatology: An Exploration of the Q-ASC Parental Report Scale in Capturing Sex Differences in Autism. *Journal of Autism and Developmental Disorders*, *48*(2), 389-403. <https://doi.org/10.1007/s10803-017-3324-9>

Oshima, F., Takahashi, T., Tamura, M., Guan, S., Seto, M., Hull, L., Mandy, W., Tsuchiya, K., & Shimizu, E. (2024). The association between social camouflage and mental health among autistic people in Japan and the UK: a cross-cultural study. *Molecular Autism*, *15*(1), 1. <https://doi.org/10.1186/s13229-023-00579-w>

Øverland, E., Andersen, P. N., Orm, S., Øie, M. G., Skogli, E. W., & Hauge, Å. L. (2024). “I Have Tried to Fit In, but I Just Want to Be Me”: Development Toward Identity Formation in Young Autistic Adults. *Autism in Adulthood*. <https://doi.org/10.1089/aut.2024.0156>

Overton, G. L., Marsà-Sambola, F., Martin, R., & Cavenagh, P. (2023). Understanding the Self-identification of Autism in Adults: a Scoping Review. *Review Journal of Autism and Developmental Disorders*. <https://doi.org/10.1007/s40489-023-00361-x>

Parish-Morris, J. (2019). Seeing the unseen realities of autism. *The Lancet Psychiatry*, *6*(9), 718-719. <https://doi.org/10.1016/s2215-0366(19)30295-0>

Parish-Morris, J., Liberman, M. Y., Cieri, C., Herrington, J. D., Yerys, B. E., Bateman, L., Donaher, J., Ferguson, E., Pandey, J., & Schultz, R. T. (2017). Linguistic camouflage in girls with autism spectrum disorder. *Molecular Autism*, *8*(1). <https://doi.org/10.1186/s13229-017-0164-6>

Pearson, A., & Rose, K. (2021). A Conceptual Analysis of Autistic Masking: Understanding the Narrative of Stigma and the Illusion of Choice. *Autism in Adulthood*. <https://doi.org/10.1089/aut.2020.0043>

Pearson, A., Rose, K., & Rees, J. (2022). ‘I felt like I deserved it because I was autistic’: Understanding the impact of interpersonal victimisation in the lives of autistic people. *Autism*, *27*(2), 500-511. <https://doi.org/10.1177/13623613221104546>

Pellicano, E., & den Houting, J. (2021). Annual Research Review: Shifting from ‘normal science’ to neurodiversity in autism science. *Journal of Child Psychology and Psychiatry*, *63*(4). <https://doi.org/10.1111/jcpp.13534>

Pellicano, E., Fatima, U., Hall, G., Heyworth, M., Lawson, W., Lilley, R., Mahony, J., & Stears, M. (2022). A capabilities approach to understanding and supporting autistic adulthood. *Nature Reviews Psychology*. <https://doi.org/10.1038/s44159-022-00099-z>

Pelton, M. K., Crawford, H., Robertson, A. E., Rodgers, J., Baron-Cohen, S., & Cassidy, S. (2020). Understanding Suicide Risk in Autistic Adults: Comparing the Interpersonal Theory of Suicide in Autistic and Non-autistic Samples. *Journal of Autism and Developmental Disorders*. <https://doi.org/10.1007/s10803-020-04393-8>

Pérez-Arqueros, M., Jamett-Cuevas, V., Pulgar-Vera, V., Santander-Gonzalez, R., Pemau, A., & Álvarez-Cabrera, P. (2025). Camouflaging and suicide behavior in adults with autism spectrum condition: A mixed methods systematic review. *Research in Autism*, *121-122*, 202540. <https://doi.org/10.1016/j.reia.2025.202540>

Perry, E., Mandy, W., Hull, L., & Cage, E. (2021). Understanding Camouflaging as a Response to Autism-Related Stigma: A Social Identity Theory Approach. *Journal of Autism and Developmental Disorders*. <https://doi.org/10.1007/s10803-021-04987-w>

Petrolini, V., Rodríguez-Armendariz, E., & Vicente, A. (2023). Autistic camouflaging across the spectrum. *New Ideas in Psychology*, *68*, 100992. <https://doi.org/10.1016/j.newideapsych.2022.100992>

Phillips, S. (2024). ‘Does that mean you will be violent?’: A qualitative exploration into Autistic women’s experiences of relationships with lecturers at university. *Autism*, 13623613241264887. <https://doi.org/10.1177/13623613241264887>

Porricelli, D., Happé, F., & Zahn, R. (2024). Individual differences in autonomy and sociotropy in relation to autistic traits, camouflaging and interpersonal functioning. *Personality and Individual Differences*, *227*, 112715. <https://doi.org/10.1016/j.paid.2024.112715>

Pryke-Hobbes, A., Davies, J., Heasman, B., Livesey, A., Walker, A., Pellicano, E., & Remington, A. (2023). The workplace masking experiences of autistic, non-autistic neurodivergent and neurotypical adults in the UK. *PLoS ONE*, *18*(9), e0290001. <https://doi.org/10.1371/journal.pone.0290001>

Pyszkowska, A. (2024). It is More Anxiousness than Role-playing: Social Camouflaging Conceptualization Among Adults on the Autism Spectrum Compared to Persons with Social Anxiety Disorder. *Journal of Autism and Developmental Disorders*. <https://doi.org/10.1007/s10803-024-06416-0>

Quigley, E., O’Hanlon, M., Brandes, M., Kennedy, R., & Gavin, B. (2024). Neurodiversity and third-level education: A lacuna between the strength-based paradigm shift and the lived experience. *Neurodiversity*, *2*, 27546330241277427. <https://doi.org/10.1177/27546330241277427>

Radulski, E. M. (2022). Conceptualising Autistic Masking, Camouflaging, and Neurotypical Privilege: Towards a Minority Group Model of Neurodiversity. *Human Development*, *66*(2), 113-127. <https://doi.org/10.1159/000524122>

Ratto, A. B., Kenworthy, L., Yerys, B. E., Bascom, J., Wieckowski, A. T., White, S. W., Wallace, G. L., Pugliese, C., Schultz, R. T., Ollendick, T. H., Scarpa, A., Seese, S., Register-Brown, K., Martin, A., & Anthony, L. G. (2018). What About the Girls? Sex-Based Differences in Autistic Traits and Adaptive Skills. *Journal of Autism and Developmental Disorders*, *48*(5), 1698-1711. <https://doi.org/10.1007/s10803-017-3413-9>

Raymaker, D. M., Teo, A. R., Steckler, N. A., Lentz, B., Scharer, M., Delos Santos, A., Kapp, S. K., Hunter, M., Joyce, A., & Nicolaidis, C. (2020). “Having All of Your Internal Resources Exhausted Beyond Measure and Being Left with No Clean-Up Crew”: Defining Autistic Burnout. *Autism in Adulthood*, *2*(2), 132-143. <https://doi.org/10.1089/aut.2019.0079>

Remnélius, K. L., Neufeld, J., Isaksson, J., & Bölte, S. (2024). Does Camouflaging Cause Reduced Quality of Life? A Co-Twin Control Study. *Journal of Autism and Developmental Disorders*. <https://doi.org/10.1007/s10803-024-06583-0>

Rhodes, S. M., Eaton, C. B., Oldridge, J., Rodgers, J., Chan, S., Skouta, E., McKechanie, A. G., Mackie, L., & Stewart, T. M. (2023). Lived experiences of depression in autistic children and adolescents: A qualitative study on child and parent perspectives. *Research in Developmental Disabilities*, *138*, 104516. <https://doi.org/10.1016/j.ridd.2023.104516>

Richdale, A. L., Morris, E. M. J., & Lawson, L. P. (2025). Suicidality in Autistic Adolescents and Adults: Sleep the Unexplored Connection? *Autism Research*, *n/a*(n/a). <https://doi.org/10.1002/aur.3306>

Ridgway, K., Cooke, K., Demmer, D. H., Hooley, M., Westrupp, E., & Stokes, M. A. (2024). Camouflaging Autism in Pursuit of Friendship and Intimate Relationships: A Systematic Review. *Autism in Adulthood*. <https://doi.org/10.1089/aut.2023.0160>

Riebel, M., Bureau, R., Rohmer, O., Clément, C., & Weiner, L. (2025). Self-compassion as an antidote to self-stigma and shame in autistic adults. *Autism*, 13623613251316965. <https://doi.org/10.1177/13623613251316965>

Riebel, M., Krasny-Pacini, A., Manolov, R., Rohmer, O., & Weiner, L. (2024). Compassion focused therapy for self-stigma and shame in autism: a single case pre-experimental study. *Frontiers in Psychiatry*, *14*. <https://doi.org/10.3389/fpsyt.2023.1281428>

Rippon, G. (2024). Differently different?: A commentary on the emerging social cognitive neuroscience of female autism. *Biology of Sex Differences*, *15*(1), 49. <https://doi.org/10.1186/s13293-024-00621-3>

Rivera, R. A., & Bennetto, L. (2023). Applications of identity-based theories to understand the impact of stigma and camouflaging on mental health outcomes for autistic people. *Front Psychiatry*, *14*, 1243657. <https://doi.org/10.3389/fpsyt.2023.1243657>

Robinson, E., & Crane, L. (2025). Examining the impact of drama-based sessions on the social emotional well-being of Autistic pupils in a special school: A multi-informant qualitative study. *British Journal of Special Education*, *n/a*(n/a). <https://doi.org/10.1111/1467-8578.70000>

Robinson, E., Hull, L., & Petrides, K. V. (2020). Big Five model and trait emotional intelligence in camouflaging behaviours in autism. *Personality and Individual Differences*, *152*, 109565. <https://doi.org/10.1016/j.paid.2019.109565>

Ross, A., Grove, R., & McAloon, J. (2022). The relationship between camouflaging and mental health in autistic children and adolescents. *Autism Research*, *16*(1). <https://doi.org/10.1002/aur.2859>

Rynkiewicz, A., Janas-Kozik, M., & Slopien, A. (2019). Girls and women with autism. *Psychiatria Polska*, *53*(4), 737-752. <https://doi.org/10.12740/PP/OnlineFirst/95098>

Rynkiewicz, A., Schuller, B., Marchi, E., Piana, S., Camurri, A., Lassalle, A., & Baron-Cohen, S. (2016). An investigation of the ‘female camouflage effect’ in autism using a computerized ADOS-2 and a test of sex/gender differences. *Molecular Autism*, *7*(1). <https://doi.org/10.1186/s13229-016-0073-0>

Sánchez-Pedroche, A., Aguilar-Mediavilla, E., Valera-Pozo, M., Adrover-Roig, D., & Valverde-Gómez, M. (2025). A preliminary study on the relationship between symptom severity and age of diagnosis in females versus males with autistic spectrum disorder. *Frontiers in Psychology*, *16*. <https://doi.org/10.3389/fpsyg.2025.1472646>

Sasson, N. J., & Morrison, K. E. (2019). First impressions of adults with autism improve with diagnostic disclosure and increased autism knowledge of peers. *Autism*, *23*(1), 50-59. <https://doi.org/10.1177/1362361317729526>

Savard, L. B., Prelock, P. A., Medeiros, S., & Lodestone, A. Z. (2024). Empowering Autistic Youth and Adults to Advocate for Mental Health and Social Connection Goals as they Transition to Adulthood. *Topics in Language Disorders*, *44*(4). <https://journals.lww.com/topicsinlanguagedisorders/fulltext/2024/10000/empowering_autistic_youth_and_adults_to_advocate.6.aspx>

Scheerer, N. E., Aime, H., Boucher, T., & Iarocci, G. (2020). The Association Between Self-Reported Camouflaging of Autistic Traits and Social Competence in Nonautistic Young Adults. *Autism in Adulthood*. <https://doi.org/10.1089/aut.2019.0062>

Scheerer, N. E., Ng, C. S. M., Gurba, A. N., McNair, M. L., Lerner, M. D., & Hargreaves, A. (2024). Editorial: Break the stigma: autism. *Frontiers in Psychiatry*, *15*. <https://doi.org/10.3389/fpsyt.2024.1513447>

Schiltz, H. K., Williams, Z. J., Zheng, S., Kaplan-Kahn, E. A., Morton, H. E., Rosenau, K. A., Nicolaidis, C., Sturm, A., Lord, C., & Autism, P. (2024). Measurement matters: A commentary on the state of the science on patient reported outcome measures (PROMs) in autism research. *Autism Research*. <https://doi.org/10.1002/aur.3114>

Schneid, I., & Raz, A. E. (2020). The mask of autism: Social camouflaging and impression management as coping/normalization from the perspectives of autistic adults. *Social Science & Medicine*, *248*, 112826. <https://doi.org/10.1016/j.socscimed.2020.112826>

Schoondermark, F., Spek, A., & Kiep, M. (2024). Evaluating an Autistic Burnout Measurement in Women. *Journal of Autism and Developmental Disorders*. <https://doi.org/10.1007/s10803-024-06438-8>

Schuck, R. K., Flores, R. E., & Fung, L. K. (2019). Brief Report: Sex/Gender Differences in Symptomology and Camouflaging in Adults with Autism Spectrum Disorder. *Journal of Autism and Developmental Disorders*, *49*(6), 2597-2604. <https://doi.org/10.1007/s10803-019-03998-y>

Schuck, R. K., Geng, A., Doss, Y., Lin, F., Crousore, H., Baiden, K. M. P., Dwyer, P., Williams, Z. J., & Wang, M. (2024). A qualitative investigation into autistic adults’ perspectives on intervention goals for autistic children. *Neurodiversity*, *2*, 27546330241266718. <https://doi.org/10.1177/27546330241266718>

Sedgewick, F., Crane, L., Hill, V., & Pellicano, E. (2019). Friends and Lovers: The Relationships of Autistic and Neurotypical Women. *Autism in Adulthood*, *1*(2), 112-123. <https://doi.org/10.1089/aut.2018.0028>

Sedgewick, F., Hill, V., & Pellicano, E. (2019). ‘It’s different for girls’: Gender differences in the friendships and conflict of autistic and neurotypical adolescents. *Autism*, *23*(5), 1119-1132. <https://doi.org/10.1177/1362361318794930>

Seers, K., & Hogg, R. (2022). “Fake it ‘till you make it”: Authenticity and wellbeing in late diagnosed autistic women. *Feminism & Psychology*, 09593535221101455. <https://doi.org/10.1177/09593535221101455>

Shalit, L., Elefant, C., & Roginsky, E. (2024). Exploring music in the everyday lives of autistic women: An Interpretative Phenomenological Analysis. *Nordic Journal of Music Therapy*, 1-20. <https://doi.org/10.1080/08098131.2024.2396105>

Shaw, S. C. K., Doherty, M., & Anderson, J. L. (2023). The experiences of autistic medical students: A phenomenological study. *Medical Education*, *57*(10), 971-979. <https://doi.org/10.1111/medu.15119>

Simcoe, S. M., Gilmour, J., Garnett, M. S., Attwood, T., Donovan, C., & Kelly, A. B. (2023). Are there gender-based variations in the presentation of Autism amongst female and male children? *Journal of Autism and Developmental Disorders*, *53*(9), 3627-3635. <https://doi.org/10.1007/s10803-022-05552-9>

Smith, O., & Jones, S. C. (2020). ‘Coming Out’ with Autism: Identity in People with an Asperger’s Diagnosis After DSM-5. *Journal of Autism and Developmental Disorders*, *50*(2), 592-602. <https://doi.org/10.1007/s10803-019-04294-5>

Somerville, M., MacPherson, S. E., & Fletcher-Watson, S. (2023). The Associations Between Camouflaging, Autistic Traits, and Mental Health in Nonautistic Adults. *Autism in Adulthood*. <https://doi.org/10.1089/aut.2023.0018>

Stark, I., Rast, J. E., Lundberg, M., Döring, N., Ohlis, A., Idring Nordström, S., Rai, D., & Magnusson, C. (2024). Completion of Upper Secondary Mainstream School in Autistic Students in Sweden. *Journal of Autism and Developmental Disorders*. <https://doi.org/10.1007/s10803-024-06470-8>

Stockwell, K. M., Bottini, S., Jaswal, V. K., & Gillis, J. M. (2020). Brief Report: Social Behavior and Special Interests in the Stigmatization of Autistic College Students. *Journal of Autism and Developmental Disorders*. <https://doi.org/10.1007/s10803-020-04769-w>

Strang, J. F., Van Der Miesen, A. I., Caplan, R., Hughes, C., Davanport, S., & Lai, M.-C. (2020). Both sex- and gender-related factors should be considered in autism research and clinical practice. *Autism*, *24*(3), 539-543. <https://doi.org/10.1177/1362361320913192>

Stroud, J., Rice, C., Orsini, A., Schlosser, M., Lee, J., Mandy, W., & Kamboj, S. K. (2025). Perceived changes in mental health and social engagement attributed to a single psychedelic experience in autistic adults: results from an online survey. *Psychopharmacology*, *242*(2), 373-387. <https://doi.org/10.1007/s00213-024-06685-8>

Sturrock, A., Guest, H., Hanks, G., Bendo, G., Plack, C. J., & Gowen, E. (2022). Chasing the conversation: Autistic experiences of speech perception. *Autism & Developmental Language Impairments*, *7*, 23969415221077532. <https://doi.org/10.1177/23969415221077532>

Sullivan, J. (2024). Organisational neurotypicalness: fighting unintentional ableism in working spaces. *Disability & Society*, 1-4. <https://doi.org/10.1080/09687599.2024.2373769>

Summerill, J., & Summers, S. J. (2025). The consequences of social camouflaging in autistic adults: A systematic review. *Research in Autism*, *121-122*, 202556. <https://doi.org/10.1016/j.reia.2025.202556>

Syharat, C. M., Hain, A., Zaghi, A. E., Gabriel, R., & Berdanier, C. G. P. (2023). Experiences of neurodivergent students in graduate STEM programs. *Frontiers in Psychology*, *14*. <https://doi.org/10.3389/fpsyg.2023.1149068>

Tafolla, M., Singer, H., & Lord, C. (2025). Autism Spectrum Disorder Across the Lifespan. *Annual Review of Clinical Psychology*. <https://doi.org/10.1146/annurev-clinpsy-081423-031110>

Tamilson, B., Eccles, J. A., & Shaw, S. C. K. (2024). The experiences of autistic adults who were previously diagnosed with borderline or emotionally unstable personality disorder: A phenomenological study. *Autism*, 13623613241276073. <https://doi.org/10.1177/13623613241276073>

Tamura, M., Cage, E., Perry, E., Hongo, M., Takahashi, T., Seto, M., Shimizu, E., & Oshima, F. (2024). Understanding Camouflaging, Stigma, and Mental Health for Autistic People in Japan. *Autism in Adulthood*. <https://doi.org/10.1089/aut.2023.0035>

Teunisse, J.-P., & De Gelder, B. (2001). Impaired Categorical Perception of Facial Expressions in High-Functioning Adolescents with Autism. *Child Neuropsychology*, *7*(1), 1-14. <https://doi.org/10.1076/chin.7.1.1.3150>

Thompson-Hodgetts, S., Labonte, C., Mazumder, R., & Phelan, S. (2020). Helpful or harmful? A scoping review of perceptions and outcomes of autism diagnostic disclosure to others. *Research in Autism Spectrum Disorders*, *77*. <https://doi.org/10.1016/j.rasd.2020.101598>

Tien, I., Pearson, A., Sozeri, S., & Seers, K. (2025). “Only Boys Can be Autistic”: A Qualitative Exploration of Gender Stereotype and Socialization on the Diagnostic Journey. *Autism in Adulthood*. <https://doi.org/10.1089/aut.2024.0178>

Tien, I., Wolpe, S., Pearson, A., & Seers, K. (2025). “Creating a socially acceptable version of myself”: A qualitative thematic analysis of the female and nonbinary experience of navigating the autism diagnostic system. *Neurodiversity*, *3*, 27546330241306380. <https://doi.org/10.1177/27546330241306380>

Tierney, S., Burns, J., & Kilbey, E. (2016). Looking behind the mask: Social coping strategies of girls on the autistic spectrum. *Research in Autism Spectrum Disorders*, *23*, 73-83. <https://doi.org/10.1016/j.rasd.2015.11.013>

Toda, S., Tsushima, S., Takashio, O., Kikuchi, M., Ohta, H., Nagasawa, T., Iwanami, A., & Ohashi, Y. (2024). The repressed life of adult female patients with mild ADHD. *Frontiers in Psychiatry*, *15*. <https://doi.org/10.3389/fpsyt.2024.1418698>

Tomas, V., Kingsnorth, S., Kirsh, B., Anagnostou, E., & Lindsay, S. (2022). Using the COM-B Model and Theoretical Domains Framework to Understand Workplace Disclosure Experiences, Influencers, and Needs Among Autistic Young Adults. *Journal of Autism and Developmental Disorders*. <https://doi.org/10.1007/s10803-022-05766-x>

Tomlinson, C., Bond, C., & Hebron, J. (2020). The school experiences of autistic girls and adolescents: a systematic review. *European Journal of Special Needs Education*, *35*(2), 203-219. <https://doi.org/10.1080/08856257.2019.1643154>

Treweek, C., Wood, C., Martin, J., & Freeth, M. (2019). Autistic people’s perspectives on stereotypes: An interpretative phenomenological analysis. *Autism*, *23*(3), 759-769. <https://doi.org/10.1177/1362361318778286>

Trundle, G., Jones, K. A., Ropar, D., & Egan, V. (2022). The forensic implications of camouflaging: a study into victimisation and offending associated with autism and pathological demand avoidance. *Advances in Autism*. <https://doi.org/10.1108/aia-02-2022-0006>

Trunk, P., Choi, B., & Rowe, M. L. (2024). Surveying Autistic Mothers About Their Parenting Practices Related to Children’s Early Language Development. *Autism in Adulthood*. <https://doi.org/10.1089/aut.2024.0138>

Tubío-Fungueiriño, M., Cruz, S., Sampaio, A., Carracedo, A., & Fernández-Prieto, M. (2020). Social Camouflaging in Females with Autism Spectrum Disorder: A Systematic Review. *Journal of Autism and Developmental Disorders*. <https://doi.org/10.1007/s10803-020-04695-x>

Underhill, J. C., Clark, J., Hansen, R. S., & Adams, H. (2024). Exploring Autistic College Students' Perceptions and Management of Peer Stigma: An Interpretative Phenomenological Analysis. *Journal of Autism and Developmental Disorders*, *54*(3), 1130-1142. <https://doi.org/10.1007/s10803-022-05867-7>

Underhill, J. C., Ledford, V., & Adams, H. (2019). Autism stigma in communication classrooms: exploring peer attitudes and motivations toward interacting with atypical students. *Communication Education*, *68*(2), 175-192. <https://doi.org/10.1080/03634523.2019.1569247>

Urbaniak, K., & D’Amico, M. (2024). Disability barriers autistic girls face in secondary education: A systematic review. *Autism*, 13623613241294189. <https://doi.org/10.1177/13623613241294189>

van der Putten, W. J., Mol, A. J. J., Groenman, A. P., Radhoe, T. A., Torenvliet, C., van Rentergem, J. A. A., & Geurts, H. M. (2024). Is camouflaging unique for autism? A comparison of camouflaging between adults with autism and ADHD. *Autism Research*, *17*(4), 812-823. <https://doi.org/10.1002/aur.3099>

van der Putten, W. J., Mol, A. J. J., Radhoe, T. A., Torenvliet, C., van Rentergem, J. A. A., Groenman, A. P., & Geurts, H. M. (2023). The relationship between camouflaging and mental health: Are there differences among subgroups in autistic adults? *Autism*, 13623613231185402. <https://doi.org/10.1177/13623613231185402>

van der Putten, W. J., van Rentergem, J. A. A., Radhoe, T. A., Torenvliet, C., Groenman, A. P., Mol, A. J. J., & Geurts, H. M. (2023). How to measure camouflaging? A conceptual replication of the validation of the Camouflaging Autistic Traits Questionnaire in Dutch adults. *Research in Autism Spectrum Disorders*, *100*, 102072. <https://doi.org/10.1016/j.rasd.2022.102072>

van Dijk, S., Peters-Scheffer, N., & Didden, R. (2024). “I Know it’s Good to Do it”: A Qualitative Study Exploring the Perspective of Autistic Men on Social Motivation. *Journal of Autism and Developmental Disorders*. <https://doi.org/10.1007/s10803-024-06651-5>

Venkatesan, S., & Tolani, P. (2024). Concealed and compromised: masking, autism and graphic medicine. *Journal of Graphic Novels and Comics*, 1-18. <https://doi.org/10.1080/21504857.2024.2426557>

Venter, F., Morelli, J., & Erasmus, E. (2022). Understanding the lived music listening experiences of adults on the autism spectrum. *Psychology of Music*, 03057356221126201. <https://doi.org/10.1177/03057356221126201>

Walsh, M. J. M., Pagni, B., Monahan, L., Delaney, S., Smith, C. J., Baxter, L., & Braden, B. B. (2023). Sex-related brain connectivity correlates of compensation in adults with autism: insights into female protection. *Cerebral Cortex*, *33*(2), 316-329. <https://doi.org/10.1093/cercor/bhac069>

Weiner, L., Bemmouna, D., Costache, M. E., & Martz, E. (2025). Dialectical Behavior Therapy in Autism. *Current Psychiatry Reports*. <https://doi.org/10.1007/s11920-025-01596-7>

Weiner, L., Costache, M. E., Bemmouna, D., Rabot, J., Weibel, S., Dubreucq, M., Dubreucq, J., & Coutelle, R. (2023). Emotion dysregulation is heightened in autistic females: A comparison with autistic males and borderline personality disorder. *Women's Health*, *19*, 17455057231174763. <https://doi.org/10.1177/17455057231174763>

White, L. C. J., Ixqe, K. V., Goodall, K., & Gillespie-Smith, K. (2024). Minority Stress, Camouflaging, and Mental Health Outcomes in Transgender and/or Non-binary Autistic Adults. *Autism in Adulthood*. <https://doi.org/10.1089/aut.2023.0151>

White, R., Barreto, M., Harrington, J., Kapp, S. K., Hayes, J., & Russell, G. (2020). Is disclosing an autism spectrum disorder in school associated with reduced stigmatization? *Autism*, *24*(3), 744-754. <https://doi.org/10.1177/1362361319887625>

Wicherkiewicz, F., & Gambin, M. (2024). Relations Between Social Camouflaging, Life Satisfaction, and Depression Among Polish Women with ADHD. *Journal of Autism and Developmental Disorders*. <https://doi.org/10.1007/s10803-024-06410-6>

Williams, Z. J. (2022). Commentary: The construct validity of 'camouflaging' in autism: psychometric considerations and recommendations for future research - reflection on Lai et al. (2020). *J Child Psychol Psychiatry*, *63*(1), 118-121. <https://doi.org/10.1111/jcpp.13468>

Wiskerke, J., Stern, H., & Igelström, K. (2018). *Camouflaging of repetitive movements in autistic female and transgender adults*. Cold Spring Harbor Laboratory. <https://dx.doi.org/10.1101/412619>

Wood-Downie, H., Wong, B., Kovshoff, H., Mandy, W., Hull, L., & Hadwin, J. A. (2020). Sex/Gender Differences in Camouflaging in Children and Adolescents with Autism. *Journal of Autism and Developmental Disorders*. <https://doi.org/10.1007/s10803-020-04615-z>

Wood‐Downie, H., Wong, B., Kovshoff, H., Cortese, S., & Hadwin, J. A. (2021). Research Review: A systematic review and meta‐analysis of sex/gender differences in social interaction and communication in autistic and nonautistic children and adolescents. *Journal of Child Psychology & Psychiatry*, *62*(8), 922-936. <https://doi.org/10.1111/jcpp.13337>

Yau, N., Anderson, S., & Smith, I. C. (2023). How is psychological wellbeing experienced by autistic women? Challenges and protective factors: A meta-synthesis. *Research in Autism Spectrum Disorders*, *102*, 102101. <https://doi.org/10.1016/j.rasd.2022.102101>

Young, H., Oreve, M. J., & Speranza, M. (2018). Clinical characteristics and problems diagnosing autism spectrum disorder in girls. *Archives de Pédiatrie*, *25*(6), 399-403. <https://doi.org/10.1016/j.arcped.2018.06.008>

Zakai-Mashiach, M. (2023). “I Was Like a Bird Without Wings”: Autistic Women’s Retrospective Experiences in General Schools. *Journal of Autism and Developmental Disorders*, *53*(11), 4258-4270. <https://doi.org/10.1007/s10803-022-05717-6>

Zakai-Mashiach, M. (2025). I was hanging in the air and holding on tight. It’s exhausting: An autistic perspective on their journey through the general school system. *Autism*, 13623613241310295. <https://doi.org/10.1177/13623613241310295>

Zener, D. (2019). Journey to diagnosis for women with autism. *Advances in Autism*, *5*(1), 2-13. <https://doi.org/10.1108/aia-10-2018-0041>

Zhang, F., & Colizzi, M. (2025). Editorial: Case reports in autism: 2023. *Frontiers in Psychiatry*, *16*. <https://doi.org/10.3389/fpsyt.2025.1563977>

Zhou, K., & Kim, J. (2024). Unraveling the Path from Autism Acceptance to Quality of Life in Autistic Transition-Age Youths: A Parallel Mediation Model. *Autism in Adulthood*. <https://doi.org/10.1089/aut.2023.0186>

Zhuang, S., Bougoure, M., Leong, D.-J., Dean, L., Reddrop, S., Naragon-Gainey, K., Maybery, M., Tan, D. W., & Magiati, I. (2024). Examining an integrated path model of psychological and sociocultural predictors of camouflaging in autistic adults. *Autism*, 13623613241262110. <https://doi.org/10.1177/13623613241262110>

Zhuang, S., Tan, D. W., Reddrop, S., Dean, L., Maybery, M., & Magiati, I. (2023). Psychosocial factors associated with camouflaging in autistic people and its relationship with mental health and well-being: A mixed methods systematic review. *Clinical Psychology Review*, *105*, 102335. <https://doi.org/10.1016/j.cpr.2023.102335>

Zolyomi, A., Begel, A., Waldern, J. F., Tang, J., Barnett, M., Cutrell, E., McDuff, D., Andrist, S., & Morris, M. R. (2019). Managing Stress: The Needs of Autistic Adults in Video Calling. *Proceedings of the ACM on Human-Computer Interaction*, *3*(CSCW), 1-29. <https://doi.org/10.1145/3359236>
